# Cucumber Mosaic Virus Infection in *Arabidopsis*: A Conditional Mutualistic Symbiont?

**DOI:** 10.3389/fmicb.2021.770925

**Published:** 2022-01-07

**Authors:** Hideki Takahashi, Midori Tabara, Shuhei Miyashita, Sugihiro Ando, Shuichi Kawano, Yoshinori Kanayama, Toshiyuki Fukuhara, Richard Kormelink

**Affiliations:** ^1^Graduate School of Agricultural Science, Tohoku University, Sendai, Japan; ^2^Department of Applied Biological Sciences, Tokyo University of Agriculture and Technology, Fuchu, Japan; ^3^Ritsumeikan Global Innovation Research Organization, Ritsumeikan University, Kusatsu, Japan; ^4^Graduate School of Informatics and Engineering, The University of Electro-Communications, Chofu, Japan; ^5^Laboratory of Virology, Department of Plant Sciences, Wageningen University and Research, Wageningen, Netherlands

**Keywords:** Argonaute 4 (AGO4), cucumber mosaic virus (CMV), CMV 2b protein, cytosine hypomethylation, defective-interfering RNA, epigenetics, persistent infection, neo-virology

## Abstract

A cucumber mosaic virus isolate, named Ho [CMV(Ho)], was isolated from a symptomless *Arabidopsis halleri* field sample containing low virus titers. An analysis of CMV(Ho) RNA molecules indicated that the virus isolate, besides the usual cucumovirus tripartite RNA genome, additionally contained defective RNA3 molecules and a satellite RNA. To study the underlying mechanism of the persistent CMV(Ho) infection in perennial *A. halleri*, infectious cDNA clones were generated for all its genetic elements. CMV, which consists of synthetic transcripts from the infectious tripartite RNA genomes, and designated CMV(Ho)tr, multiplied in *A. halleri* and annual *Arabidopsis thaliana* Col-0 to a similar level as the virulent strain CMV(Y), but did not induce any symptoms in them. The response of Col-0 to a series of reassortant CMVs between CMV(Ho)tr and CMV(Y) suggested that the establishment of an asymptomatic phenotype of CMV(Ho) infection was due to the *2b* gene of CMV RNA2, but not due to the presence of the defective RNA3 and satellite RNA. The accumulation of CMV(Ho) 2b protein tagged with the FLAG epitope (2b.Ho-FLAG) in *2b.Ho-FLAG*-transformed Col-0 did not induce any symptoms, suggesting a 2b-dependent persistency of CMV(Ho)tr infection in *Arabidopsis*. The 2b protein interacted with Argonaute 4, which is known to regulate the cytosine methylation levels of host genomic DNA. Whole genomic bisulfite sequencing analysis of CMV(Ho)tr- and mock-inoculated Col-0 revealed that cytosine hypomethylation in the promoter regions of 82 genes, including two genes encoding transcriptional regulators (*DOF1.7* and *CBP1*), was induced in response to CMV(Ho)tr infection. Moreover, the increased levels of hypomethylation in the promoter region of both genes, during CMV(Ho)tr infection, were correlated with the up- or down-regulation of their expression. Taken altogether, the results indicate that during persistent CMV(Ho) infection in *Arabidopsis*, host gene expression may be epigenetically modulated resulting from a 2b-mediated cytosine hypomethylation of host genomic DNA.

## Introduction

Surveys of viral infections in wild perennial plants have revealed the existence of diverse and variable virus communities (Alexander, 2010; Malmstrom et al., 2011; Owens et al., 2012; Roossinck, 2012; Seabloom et al., 2015; Malmstrom and Alexander, 2016; Takahashi et al., 2019). Despite these findings, most knowledge on viral pathosystems have been collected from studying symptomatic infections of cultivated plant species (Wren et al., 2006). More recent studies using metagenomic approaches have indicated that asymptomatic (latent) or persistent viral infections of host plants or viral infections that induce very mild symptoms, thereby not inducing a lethal phenotype, might be a much more common event in nature than initially thought (Kreuze et al., 2009; Barba et al., 2014; Stobbe and Roossinck, 2014; Kamitani et al., 2016; Pooggin, 2018; Zhang et al., 2018; Susi et al., 2019; Tabara et al., 2021). Moreover, the integration of nucleotide sequences partially or fully corresponding to viral genes into plant genomic DNA has been found in several host plants, suggesting that viruses and host plants might have lived together symbiotically during their evolution (Holmes, 2011; Teycheney and Geering, 2011; Feschotte and Gilbert, 2012; Aiewsakun and Katzourakis, 2015; Kuriyama et al., 2020).

In general, the status of animal virus infection is categorized according to the existence and replication level of virus and development of symptoms in host organisms. Two of the classifications of infection status are overt infection and subclinical infection, which are judged by the presence or absence of symptoms. The former has symptoms and the latter does not, but viruses may be maintained in host organisms. In addition, there are acute and persistent infections classification according to the existence period of the pathogen in host organisms; persistent infection is further divided into latent infection, chronic infection, and late infection. Virus latency is the ability of a pathogenic virus to lie latent within a host cell. Latency is the phase in certain virus life cycles in which, after initial infection, proliferation of virus particles stops. However, the viral genome is not eradicated. The virus can reactivate and begin to produce progeny virus.

In the case of many wild plants, viral infection is maintained for a long period, but the plants are commonly symptomless or exhibit very mild symptoms, thereby not inducing a lethal phenotype in nature, even if the virus restarts to replicate, when environmental conditions, e.g., natural temperature, become appropriate for viral multiplication during four seasons. On the other hand, when the environmental conditions become worse, viral replication seems to decrease or cease. Thus, in some cases, the categorization of the status of plant viral infection in host plants is not always in agreement with animal viral infection. In this study, we would like to define such status of natural viral infection in wild host plants as “persistent infection.”

Although persistent viral infections and viral gene integration in host plants may be common events in the natural ecosystem, information on the influence of viral infection and integrated endogenous viral elements on host plants is still limited (Takahashi et al., 2019). Roossinck (2011) has indicated the beneficial effects of viral infections on host plants such as conferring drought or cold tolerance (Xu et al., 2008). Although several metabolites and processes, including osmoprotectants and abscisic acid-mediated signaling, seem to be associated with these tolerances (Xu et al., 2008; Westwood et al., 2012), the underlying molecular mechanisms leading to those beneficial effects on the plant host have not been elucidated. Recently, viral infections were shown to have a positive effect on plant reproduction by increasing pollinator preference (Groen et al., 2016). Increased buzz pollination of virus-infected plants raises their seed yield to quantities comparable to those of mock-inoculated plants. The emission profiles of volatile organic compounds from virus-infected *Solanum lycopersicum* and *Arabidopsis thaliana* seem to be associated with an alteration in the foraging behavior of bumblebees (*Bombus terrestris*), thereby increasing buzz pollination (Groen et al., 2016). In another recent study, the behavior of aphids, which are vectors of many plant viruses, was altered on plants that did not show any symptoms but were persistently infected with a virus (Safari et al., 2019). Plant viruses may also influence the susceptibility/preference of plants to different biotic stressors and provide obvious beneficial physiological traits for plant life without visible symptoms, while the underlying mechanisms causing those effects have not been studied widely (Nakatsukasa-Akune et al., 2005; Pagán et al., 2007, 2008; van Molken et al., 2012; Shapiro et al., 2013; Hily et al., 2014, 2016; Harth et al., 2018; Khankhum and Valverde, 2018; Fukuhara et al., 2019).

*Arabidopsis halleri* is a perennial weed that inhabits high- and middle-altitude regions throughout Europe and Asia (Hohmann et al., 2014; Šrámková-Fuxová et al., 2017; Honjo and Kudoh, 2019). *A. halleri* is a close relative of the annual weed *A. thaliana*, but *A. halleri* is self-incompatible, whereas *A. thaliana* is self-compatible, providing a model system to elucidate the molecular evolution of self-fertilization. In addition, *A. halleri* has an ability to hyperaccumulate heavy metals; thus, it has received considerable attention as a resource for phytoremediation (Cho et al., 2003). Natural infection of *A. halleri* with some plant viruses including cucumber mosaic virus (CMV), turnip mosaic virus, and a partitivirus has been reported (Kamitani et al., 2019).

CMV has a large host range of over 1,200 plant species, and infection with various strains and isolates leads to a variety of symptoms that differ in severity (Hull, 2013; Palukaitis and García-Arenal, 2019). CMV is transmitted mainly by aphids and is spread widely in fields, thereby causing severe economic losses of crops and ornamental species. In winter, CMV can survive in perennial plants, including overwintering weeds, which provides a source of infection to crops *via* aphids in spring (Palukaitis and García-Arenal, 2019). CMV-infected perennial plants such as *A. halleri* seem to show mild symptoms that do not cause lethal damage to their growth, but are often symptomless, whereas persistent viral infection is maintained during the life cycle of *A. halleri* (Kamitani et al., 2019). Thus, once a perennial plant is infected with a virus, it may maintain the virus for a long period of time.

CMV is a well-characterized isometric particle virus that has three positive single-stranded RNA segments: RNA1, RNA2, and RNA3. RNA1 and RNA2 encode the components of virus replicase, and RNA3 has two open reading frames (ORFs) encoding the cell-to-cell movement 3A protein and the coat protein, respectively (Palukaitis and García-Arenal, 2019). The coat protein is translated from RNA4, which is a subgenomic RNA containing ORF encoding the coat protein and transcribed from a minus strand of RNA3 in virus-infected cells. RNA2 additionally encodes a 2b protein that is expressed *via* the synthesis of a subgenomic RNA2 molecule. The 2b protein presents a viral suppressor of RNA silencing (VSR) and seems to affect host microRNA metabolism through its direct interaction with Argonaute 1 (AGO1), the core component of the RNA-induced silencing complex (Zhang et al., 2006; González et al., 2010), thereby inducing systemic symptoms in host plants. Furthermore, the 2b protein of CMV strains can bind to Argonaute 4 (AGO4) and AGO4-related small RNAs, thereby impairing AGO4 activity (Duan et al., 2012; Hamera et al., 2012) and suppressing transcriptional gene silencing mediated by small-RNA-directed DNA methylation (RdDM). Some CMV strains contain satellite RNA (sat-RNA) molecules that multiply in a CMV genomic RNA replication-dependent manner and modulate symptom expression (Palukaitis and García-Arenal, 2019).

Recently, CMV isolate Ho [CMV(Ho)] was isolated from symptomless *A. halleri* plants that were growing in a natural field in Japan. In this study, we characterized CMV(Ho), identified the viral molecules causing persistent infection, and analyzed its interaction with host proteins and its impact on the status of host genomic DNA by modulating cytosine hypomethylation levels. The obtained results revealed an important role of CMV 2b protein on an altered host cell environment under persistent infection with CMV(Ho).

## Materials and Methods

### Plants and Virus

Virus-free *A. halleri* subsp. *gemmifera* cv. AHG347 Winterclean (Miyoshi Co., Ltd. and Fujita Co., Ltd., Tokyo, Japan) (Ministry of Agriculture Forestry and Fisheries, 2012), *A. thaliana* ecotype Col-0, *Chenopodium amaranticolor*, *Vigna unguiculata*, and *Nicotiana benthamiana* were grown on a soilless mix (Metro-Mix^®^ 380; Sun Gro Horticulture, Agawam, MA) under a 14-h light (14,000 lux)/10-h dark photoperiod at 25°C in a KG-201 HL-D growth chamber (Koito, Yokohama, Japan). *N. benthamiana* was used for virus propagation and *V. unguiculata* was done for assessment of the infectivity of purified CMV.

CMV was isolated from a symptomless *A. halleri* community naturally growing on an independent area of an abandoned “Hosokura” mine in the Tohoku region of Japan through a single lesion isolation method using *C. amaranticolor* (Bhat and Rao, 2020) and named CMV(Ho). A yellowing strain of CMV [CMV(Y)] (Tomaru and Hidaka, 1960) and an Indonesian strain of CMV [CMV(B2)] (Suastika et al., 1995), showing virulence to host plants, were used as controls.

CMV(Ho)tr, a 13-reassortant CMV between CMV(Ho)tr and CMV(Y), and CMV(Ho)tr containing defective interfering RNA3 (DI-RNA3) or sat-RNA were produced by mixed infectious RNA molecules *in vitro* transcribed from corresponding cDNA to each CMV genome RNA, DI-RNA3, or sat-RNA. The information of the purified CMV, encapsulated genomic RNA molecules, and *in vitro* transcription vectors are summarized in Supplementary Table 1. The detailed procedures for construction of each *in vitro* transcription vector, *in vitro* transcription of infectious CMV RNA, and virus propagation and purification are described below.

### Construction of *in vitro* Transcription Vectors Carrying cDNA for Cucumber Mosaic Virus RNA

Total RNA was isolated from *A. halleri* infected with CMV(Ho) using RNeasy Plant Mini Kit (Qiagen GmbH, Hilden, Germany) according to the manufacturer’s instructions. cDNAs for CMV(Ho) RNA1, RNA2, and RNA3 were synthesized by RT-PCR using 5′- and 3′-CMV primer sets: *Hin*dIII + T7pro + CMV.RNA1.5′, *Bam*HI + T7pro + CMV.RNA2.5′, and *Bam*HI + T7pro + CMV.RNA3.5′, which contained the T7 promoter sequence, respectively, and CMV.RNA1.3′ + *Not*I, CMV.RNA2.3′ + *Not*I, and CMV.RNA3.3′ + *Not*I, which contained a *Not*I restriction site, respectively (Supplementary Table 3), using a PrimeScript™ II High Fidelity One Step RT-PCR Kit (Takara Bio, Shiga, Japan) according to the instruction manual and as previously described (Tian et al., 2020). All PCR products were purified using the Wizard^®^ SV Gel and PCR Clean-Up (WGPC) System (Promega, Madison, United States). cDNA for CMV RNA1 was cloned into the *Hin*dIII and *Not*I sites of pCY1-T7 (Suzuki et al., 1991), and cDNAs for CMV RNA2 and RNA3 were cloned into the *Bam*HI and *Not*I sites of pCY2-T7 or pCY3-T7 (Suzuki et al., 1991) using the In-Fusion HD Cloning System (Takara Bio) according to the standard protocol. The plasmid constructs containing each cDNA for CMV RNA1, RNA2, and RNA3 under the control of the T7 promoter were designated as pCH1-T7, pCH2-T7, and pCH3-T7, respectively (Supplementary Figure 1). The plasmid constructs of cDNA for DI-RNA3, which was cloned under the control of the T7 RNA promoter, were selected from the clones during pCH3-T7 construction. The plasmid constructs containing CMV(Ho) DI-RNA3 cDNA were designated as CMV(Ho)RNA3.DI-1 and CMV(Ho)RNA3.DI-6 (Supplementary Figure 2, equal to pCH.RNA3.DI-6-T7 in Supplementary Figure 1). The nucleotide sequences of cDNAs for CMV(Ho) RNA1, RNA2, and RNA3 in pCH1-T7, pCH2-T7, and pCH3-T7, and for CMV(Ho) RNA3.DI-1 and DI-6, were determined by Sanger sequencing using a CEQ8000 Automated DNA Sequencer (Beckman Coulter, Brea, United States) and registered in the GenBank/EMBL/DDBJ databases. For controls, pCY1-T7, pCY2-T7, and pCY3-T7, from which infectious CMV(Y) RNA1, RNA2, and RNA3 with virulence to *A. thaliana* ecotype Col-0 were *in vitro* transcribed, were used (Suzuki et al., 1991).

cDNA for CMV(Ho) sat-RNA was also synthesized from total RNA by RT-PCR. RT-PCR primers for CMV(Ho) sat-RNA, *Hin*dIII + T7pro + CMV.satRNA1.5′ and a mixture of CMV.satRNA1.3′ + *Not*I and CMV.satRNA1.3′ + *Not*I.ver.2 (Supplementary Table 3), were used. The PCR product was purified using the WGPC System (Promega) and cloned into the *Bam*HI and *Not*I sites of pCY3-T7 using the In-Fusion HD Cloning System (Takara Bio). The plasmid construct containing sat-RNA cDNA was designated as pCH.sat-T7 (Supplementary Figure 1). The nucleotide sequence of cDNA for sat-RNA in pCH.sat-T7 was determined by Sanger sequencing as well and registered in the GenBank/EMBL/DDBJ databases.

pC2(Y-H)-T7 and pC2(H-Y)-T7 (Supplementary Figure 1), in which chimeric RNA2 cDNAs were reciprocally exchanged with the ORFs encoding 2a and 2b proteins between CMV(Ho) and CMV(Y), were constructed by two-step PCR using pCH2-T7 and pCY2-T7 as previously described (Tian et al., 2020). All PCR products were purified using the WGPC System (Promega). In the first round of PCR, the 3′- and 5′-products of RNA2 cDNA, which encode 2a and 2b proteins, respectively, were amplified using CMV(Ho) or CMV(Y) cDNA as a template with two sets of primers, *Bam*HI + T7pro + CMV.RNA2.5′ and an internal reverse primer RNA2.2335-2361.R2 and an internal forward RNA2.2335-2361.F2 and CMV.RNA2.3′ + *Not*I, respectively (Supplementary Table 3). In the second round of PCR, the resulting 5′- and 3′-products of RNA2 cDNA amplified in the first round of PCR were used as templates to produce full-length chimeric RNA2 cDNA using the primers *Bam*HI + T7pro + CMV.RNA2.5′ and CMV.RNA2.3′ + *Not*I. The gel-purified RNA2 cDNA fragment was cloned into the *Hin*dIII and *Not*I sites of pCY2-T7. The nucleotide sequences of the plasmid constructs, pC2(Y-H)-T7 and pC2(H-Y)-T7, were determined by Sanger sequencing.

pCY2.2b(S77L), pCY2.2b(A21V), and pCY2.2b(A106V) (Supplementary Figure 3), in which single-nucleotide substitutions in the *2b* gene of CMV(Y) RNA2 caused a single amino acid substitution in 2b protein (A21V, S77L, or A106V), were constructed by two-step PCR using pCY2-T7. In the first round of PCR, the 3′- and 5′-products of RNA2 cDNA were amplified using pCY2-T7 as a template with two sets of primers, *Bam*HI + T7pro + CMV.RNA2.5′ and 2b.A21V.R, 2b.S77L.R, or 2b.A106V.R and CMV.RNA2.3′ + *Not*I and 2b.A21V.F, 2b.S77L.F, or 2b.A106V.F, respectively (Supplementary Table 3). The primer sets 2b.A21V.F and 2b.A21V.R, 2b.S77L.F and 2b.S77L.R, and 2b.A106V.F and 2b.A106V.R were based on the junction site for chimeric constructs and contained a single-nucleotide substitution. pCY2.2b(S77L/A106V) and pCY2.2b(A21V/S77L) (Supplementary Figure 3), in which two-nucleotide substitutions in the *2b* gene of CMV(Y) RNA2 resulted in two amino acid substitutions in 2b protein (A21V/S77L or S77L/A106V), were also constructed by two-step PCR using pCY2-T7 with the same set of primers as for single-nucleotide substitution (2b.A21V.F and 2b.A21V.R; 2b.A106V.F and 2b.A106V.R) (Supplementary Table 3) and pCY2.2b(S77L) as a template for PCR. In the second round of PCR, the resulting 5′- and 3′-products of RNA2 cDNA amplified in the first PCR were used as templates to produce full-length chimeric RNA2 cDNA using the primers *Bam*HI + T7pro + CMV.RNA2.5′ and CMV.RNA2.3′ + *Not*I (Supplementary Table 3) as previously described (Tian et al., 2020). The gel-purified RNA2 cDNA fragment was cloned into the *Bam*HI and *Not*I sites of pCY2-T7 (Takara Bio). The nucleotide sequences of the plasmid constructs containing nucleotide substitutions in the *2b* gene of RNA2, namely, pCY2.2b(S77L/A106V), pCY2.2b(A21V/S77L), pCY2.2b(S77L), pCY2.2b(A21V), and pCY2.2b(A106V) (Supplementary Figure 3), were confirmed by Sanger sequencing.

### Phylogenetic Tree Analysis of Deduced Amino Acid Sequences of the Coat Protein of Cucumber Mosaic Virus

To classify CMV(Ho), the phylogenetic relationship based on the deduced amino acid sequence of the coat protein (CP) was determined between CMV(Ho) and 11 other CMV strains: all amino acid sequences of the CP of other CMV strains were identified according to the isolate name and GenBank accession number: CMV(Y) (accession number D12499), CMV(As) (accession number Q66154), CMV(ND1) (accession number EU414785), CMV(ND2) (accession number EU414786), and CMV(Fny) (accession number D10538) in subgroup IA; CMV(M) (accession number Q00260), CMV(KS44) (accession number AJ810259), CMV(P6) (accession number Q00261), and CMV(FC) (accession number Q00259) in subgroup IB; and CMV(Q) (accession number M21464) and CMV(TN) (accession number AB176847). The amino acid sequence of the CP of peanut stunt virus (PSV) strain ER (accession number U15730) was used as an outgroup. The tree was created based on the neighbor-joining method using MEGA11 (Saitou and Nei, 1987; Tamura et al., 2021). The percentage of replicate trees, in which the associated taxa clustered together in the bootstrap test (1,000 replicates), is shown next to the branches. The tree is drawn to scale, with branch lengths (next to the branches) in the same units as those of the evolutionary distances used to infer the phylogenetic tree.

### *In vitro* Transcription of Infectious Cucumber Mosaic Virus RNA and Production of Reassortant Cucumber Mosaic Virus

pCH1-T7, pCH2-T7, pCH3-T7, pCY1-T7, pCY2-T7, pCY3-T7, pCH.RNA3.DI-6-T7, pCH.sat-T7, pC2(Y-H)-T7, and pC2(H-Y)-T7 were linearized by digestion with *Not*I and purified using the WGPC System (Promega). Each linearized plasmid DNA was transcribed *in vitro* using T7 RNA polymerase with an AmpliCap-Max™ T7 High Yield Message Maker Kit (CELLSCRIPT, Madison, United States) according to the manufacturer’s instructions. After incubation for 60 min at 37°C, the obtained RNA was used as inoculum to propagate virus: CMV(Ho)tr, CMV(Y), CMV(Ho)tr + RNA3.DI-6, and CMV(Ho)tr + sat-RNA, respectively, shown in Supplementary Table 1. Six-week-old *N. benthamiana* was inoculated with an appropriate combination of RNA transcripts, e.g., a combination of RNA transcribed from pCH1-T7, pCH2-T7, and pCH3-T7 for propagation of CMV(Ho)tr. At 7 days post-inoculation, the inoculated leaves were harvested, and virus was purified by the method as previously described (Takahashi and Ehara, 1993; Tian et al., 2020). A series of reassortant CMVs, including CMV(HYY), CMV(YHY), CMV(YHH), CMV(YYH), CMV(HYH), and CMV(HHY), and CMV(Y.HaYb.Y) and CMV(Y.YaHb.Y) carrying chimeric RNA2 with reciprocally exchanged ORFs encoding 2a and 2b proteins between CMV(Ho) and CMV(Y), shown in Supplementary Table 1, were also propagated by the same method.

For propagation of CMV carrying nucleotide substitution(s) in the *2b* gene of RNA2: CMV(2b.S77L/A106V), CMV(2b.A21V/S77L), CMV(2b.S77L), CMV(2b.A21V), and CMV(2b.A106V) (Supplementary Table 1), *Not*I-digested plasmid DNA of pCY2.2b(S77L/A106V), pCY2.2b(A21V/S77L), pCY2.2b(S77L), pCY2.2b(A21V), and pCY2.2b(A106V), respectively, were used for *in vitro* transcription of infectious RNA2. Each RNA2 transcript was used to inoculate *N. benthamiana* with CMV(Y) RNA1 and RNA3, which were *in vitro* transcribed from pCY1-T7 and pCY3-T7, respectively. At 7 days post-inoculation, virus was purified as previously described (Takahashi and Ehara, 1993; Tian et al., 2020).

The concentration of virus was adjusted at 100 μg/ml in 50 mM sodium phosphate buffer (pH 7.2). An absorbance value 0.5 at 260 nm was converted into 100 μg/ml (Noordam, 1974). Moreover, to evaluate the infectivity of each virus, 10 primary leaves of 7-day-old cowpea (*V. unguiculata*) were rub-inoculated with the aliquot of the virus solution, and ∼100 necrotic local lesions formed were confirmed.

### Virus Inoculation and Detection

Fully expanded leaves of *A. halleri* and *A. thaliana* were rub-inoculated with 100 μg/ml virus as previously described (Takahashi et al., 1994). The coat protein was detected immunologically by western blot analysis according to the standard protocol (Sambrook and Russell, 2001) using an antibody against the coat protein of CMV. The amount of coat protein was measured quantitatively by an enzyme-linked immunosorbent assay (ELISA) as previously described (Sekine et al., 2004). The distribution of virus in systemically infected plants was analyzed immunologically by a modified tissue printing method (Takahashi et al., 2002).

CMV RNA in virion or virus-inoculated leaves was detected by northern hybridization. CMV RNA was purified from CMV particles according to the method previously described (Takahashi and Ehara, 1993). Total RNA was isolated from *A. thaliana* ecotype Col-0 infected with CMV. The northern hybridization was done according to the standard protocol (Sambrook and Russell, 2001) using DIG-labeled cDNA probes, which can be specifically hybridized with CMV RNA1, RNA2, RNA3, and sat-RNA, respectively, or DIG-labeled cDNA probe of the 3′-end conserved sequence to CMV RNA1, RNA2, and RNA3. Each DIG-labeled cDNA probe was amplified by PCR using cDNA to CMV RNA1, RNA2, RNA3, or sat-RNA as a template and a set of primers as shown in Supplementary Table 2. As an internal control, rRNA on the northern blot membrane was stained by methylene blue according to the standard protocol (Sambrook and Russell, 2001).

For quantifying the full-length CMV RNA3 in CMV-inoculated leaves of the Col-0 by quantitative PCR (qPCR), total RNA (100 ng) was reverse transcribed into cDNA using a PrimeScript RT Reagent Kit with gDNA Eraser (Takara Bio) containing random hexamer primers according to the manufacturer’s instructions. qPCR for CMV RNA3 was performed in triplicate, in 20-μl reactions containing 2 μl template cDNA, a set of primers: 0.4 mM 3A-F (5′-GGCATGGCTTTCCAAGGTACCA-3′) and 0.4 mM RNA3.1077-1053R (5′-CCCTTCTCAACACGGCATCGCGTC-3′); 1 × ROX Reference Dye; and 1 × SYBR Premix Ex Taq II (Tli RNase H plus; Takara Bio) using the 7300 Real-Time PCR System (Applied Biosystems, Foster City, CA). The primer set specifically amplified a part of the full-length CMV RNA3, but it did not in either RNA3.DI-6 or sat-RNA. As an internal control, *A. thaliana ACT2* transcript levels were measured by qPCR with the primers ACT2-F1 (5′-AATCACAGCACTTGCACCA-3′) and ACT2-R1 (5′-GAGGGAAGCAAGAATGGAAC-3′). CMV RNA levels were normalized relative to the values of the constitutively expressed *ACT2* mRNA. PCR conditions and data analysis were performed as previously described (Takahashi et al., 2014).

### Construction of Transient Expression Vectors Carrying Hemagglutinin Epitope Sequence-Tagged Cucumber Mosaic Virus *2b* cDNA Under the Control of the CaMV 35S Promoter

cDNAs encoding the 2b protein of CMV(Ho) and CMV(Y) were synthesized from pCH2-T7, pCY2-T7, pCY2.2b(S77L/A106V), and pCY2.2b(A21V/S77L), respectively. The primer sets CMV.2b.F1 + 15*Nde*I and CMV(Y).2b.R1 + HA + 15*Sal*I for pRI201AN:2b.Ho and pRI201AN:2b.Y, CMV.2b.F1 + 15*Nde*I and CMV(Y).2b.A21V/S77L + HA + 15*Sal*I for pRI201AN:2b.Y(A21V/S77L), and CMV.2b.F1 + 15*Nde*I and CMV(Y).2b.S77L/A106V + HA + 15*Sal*I for pRI201AN:2b.Y(S77L/A106V), which contained the hemagglutinin (HA)-epitope sequence (deduced amino acid sequence: YPYDVPDYA) at the 3′-end of *2b* cDNA, were used for PCR (Supplementary Table 4 and Supplementary Figure 4A). The gel-purified HA sequence-tagged *2b* cDNA products were cloned into the *Nde*I and *Sal*I sites of the binary pRI201-AN vector (Takara Bio). The nucleotide sequences of the vector constructs pRI201AN:2b.Ho, pRI201AN:2b.Y, pRI201AN:2b.Y(A21V/S77L), and pRI201AN:2b.Y(S77L/A106V), carrying HA sequence-tagged *2b* cDNA, were confirmed. pRI201AN:GFP was constructed as previously described (Takahashi et al., 2012).

*Agrobacterium tumefaciens* LBA4404 (Takara Bio) was transformed with pRI201AN:2b.Ho, pRI201AN:2b.Y, pRI201AN:2b.Y(A21V/S77L), pRI201AN:2b.Y(S77L/A106V), pRI201AN:GFP, or pRI201-AN (control), by the standard protocol (Sambrook and Russell, 2001). The obtained transformants were used for transient expression in *N. benthamiana* 16c leaves by the agroinfiltration method (Haseloff et al., 1997; Ruiz et al., 1998), as previously described (Takahashi et al., 2012). Green fluorescent protein (GFP) expression in agroinfiltrated leaves of *N. benthamiana* was observed using an Illumatool LT-99D2 dual lighting system (Lightools Research, Encinitas, United States) with an LT-9470FX excitation filter (470 nm)/LT-9GFPVG emission filter (515 nm). Quantitative measurement of GFP accumulation in the agroinfiltrated leaves was conducted by ELISA according to the protocol described previously (Sekine et al., 2004). Anti-GFP polyclonal antibody (Medical and Biochemical Laboratories Co., Ltd., Tokyo Japan) was used for ELISA. Accumulated HA-tagged 2b protein was detected immunologically using a horseradish peroxidase (HRP)-labeled anti-HA monoclonal antibody (clone 3F10, dilution 1:10,000; Roche, Indianapolis, United States) according to the standard protocol (Sambrook and Russell, 2001).

### Transient and Stable Expression of p35S:2b.Ho Tagged With the FLAG Epitope Sequence at the 3′-Terminus and p35S:AGO1-10 Tagged With the HA Epitope Sequence at the 5′-Terminus

cDNAs encoding the 2b protein of CMV(Ho) and CMV(Y) RNA2 were synthesized from pCH2-T7 and pCY2-T7, respectively. The primer sets CMV.2b.F1 + 15*Nde*I and CMV(Ho).2b.R1 + FLAG + 15*Sal*I for FLAG-epitope sequence-tagged CMV(Ho)2b and CMV.2b.F1 + 15*Nde*I and CMV(Y).2b.R1 + FLAG + 15*Sal*I for FLAG-epitope sequence-tagged CMV(Y)2b, which contained the FLAG-epitope sequence (deduced amino acid sequence: DYKDDDDK) at the 3′-end of *2b* cDNA (Supplementary Table 4), were used for PCR. The gel-purified FLAG-epitope sequence-tagged *2b* cDNA fragments of CMV(Ho) and CMV(Y) were cloned into the *Nde*I and *Sal*I sites of the binary pRI201-AN vector (Takara Bio), respectively. The nucleotide sequences of vector constructs carrying FLAG-epitope sequence-tagged *2b* cDNA of CMV(Ho) and CMV(Y) (p35S:2bHo-FLAG and p35S:2bY-FLAG, respectively) (Supplementary Figure 4B) were confirmed.

cDNAs for *AGO1-10* mRNA were synthesized from total RNA isolated from *A. thaliana* ecotype Col-0 by RT-PCR. The primer sets for RT-PCR, HA-epitope sequence at the 5′-end of each forward-strand AGO sequence and each corresponding reverse-strand sequence, are shown in Supplementary Table 4. The gel-purified HA-epitope sequence-tagged *AGO1-10* cDNA products were cloned into the *Nde*I and *Sal*I sites of the binary pRI201-AN vector (Takara Bio). The resulting vector constructs were named as p35S:HA-AGO1, p35S:HA-AGO2, p35S:HA-AGO3, p35S:HA-AGO4, p35S:HA-AGO5, p35S:HA-AGO6, p35S:HA-AGO7, p35S:HA-AGO8, p35S:HA-AGO9, and p35S:HA-AGO10 (Supplementary Figure 4B). The nucleotide sequences of those vector constructs were confirmed.

*A. tumefaciens* LBA4404 (Takara Bio) was transformed with p35S:2bH-FLAG, p35S:2bY-FLAG, p35S:HA-AGO1-10, or pRI201-AN. The obtained transformants were used for the transient expression of 2b.Ho-FLAG, 2b.Y-FLAG, or each *AGO* cDNA by the agroinfiltration method in *N. benthamiana* leaves, as previously described (Takahashi et al., 2012), or to generate transgenic *A. thaliana* ecotype Col-0 constitutively expressing 2b.Ho-FLAG, 2bY-FLAG, or AGO4 cDNA.

*A. thaliana* ecotype Col-0 was transformed with the vacuum infiltration method (Bechtold and Bouchez, 1995). Transformants carrying the heterozygous transgene were screened by the growth of germinated plants on 1/2 Murashige–Skoog medium (Murashige and Skoog, 1962) containing 50 μg/ml kanamycin. Three transgenic plant lines carrying the homozygous transgene were established from the progeny of M2 and M3 generations by immunological detection of 2b.Ho-FLAG or 2b.Y-FLAG protein using an HRP-labeled anti-FLAG monoclonal antibody (clone M2, dilution 1:10,000; Sigma-Aldrich, Darmstadt, Germany) or HA-AGO4 protein using an HRP-labeled anti-HA monoclonal antibody (clone 3F10, dilution 1:10,000) according to the standard protocol (Sambrook and Russell, 2001). The resulted transformants were named as Col:35Spro.2b.Ho-FLAG, Col: 35Spro.2b.Y-FLAG, and Col: 35Spro.HA-AGO4, respectively. Three independent transgenic Col-0 lines carrying the homozygous transgenes 35S:2b.Ho-FLAG and 35S:HA-AGO4 were screened from the progeny of the crosses between Col:35Spro.2b.Ho-FLAG and Col:35Spro.HA-AGO4 by detecting the accumulation of 2b.Ho-FLAG and HA-AGO4 proteins in all progeny.

### Co-immunoprecipitation of CMV(Ho) 2b Protein With AGO Proteins

*A. tumefaciens* LBA4404 transformed with p35S:2b.Ho-FLAG, p35S:HA-AGO1-10, or pRI201-AN as a control was cultured by the procedure described previously (Takahashi et al., 2012). After centrifugation at 7,000 rpm for 10 min, the bacterial pellet was resuspended in 1 ml of 50 mM MES buffer (pH 7.2) containing 10 mM MgCl_2_. The concentration of *Agrobacterium* was determined by measurement at OD_600_. Fully expanded leaves of *N. benthamiana* were infiltrated with OD_600_ = 0.2 of *A. tumefaciens* LBA4404 transformed with p35S:2b.Ho-FLAG, p35S:HA-AGO1-10, or pRI201-AN as a control, as previously described (Takahashi et al., 2012). At 48 h after agroinfiltration, 1 g fresh weight of the infiltrated region in the leaves was sampled and homogenized in 5 ml of 25 mM Tris (pH 7.5), 10% (*v*/*v*) glycerol, 1 mM EDTA, 150 mM NaCl, 10 mM DTT, 2% (*w*/*v*) polyvinylpolypyrrolidone, and 1 × protease inhibitor cocktail (P9599; Sigma-Aldrich) on ice by mortar and pestle. A co-immunoprecipitation (Co-IP) assay using EZview™ Red Anti-HA Affinity Gels (E6779; Sigma-Aldrich) was performed according to the method reported by Moffett (2011). The 2b.Ho-FLAG protein, which was co-immunoprecipitated with HA-AGO proteins, was detected by western blot using an HRP-labeled anti-FLAG monoclonal antibody (clone M2; Sigma-Aldrich). HA-AGO proteins were done by western blot using an HRP-labeled anti-HA monoclonal antibody (clone 3F10; Roche).

To further analyze the interaction of HA-AGO4 protein with 2b.Ho-FLAG protein, 1 g of fresh weight leaves from three 4-week-old independent transgenic Col-0 lines (Col:35Spro.2b.Ho-FLAG/35Spro.HA-AGO4 #1, #2, and #3) carrying homozygous transgenes (35S:2b.Ho-FLAG and 35S:HA-AGO4) were used for the Co-IP assay, as described above (Moffett, 2011).

### Whole-Genome Bisulfite Sequencing of CMV(Ho)tr- and Mock-Inoculated Leaves of *Arabidopsis thaliana* Ecotype Col-0

Genomic DNA was isolated from CMV(Ho)tr- and mock-inoculated leaves of Col-0 plants at 7 days post-infection (dpi) using a DNeasy Plant Maxi Kit (Qiagen) according to the manufacturer’s instructions. For whole-genome bisulfite sequencing, a bisulfite-treated DNA library, in which unmethylated cytosine was converted to uracil and methylated cytosine was retained, was prepared using an Accel-NGS Methyl-Seq DNA Library Kit and an EZ DNA Methylation-Gold Kit (Zymo Research, Irvine, United States). Bisulfite conversion, denaturation, and adaptase reaction steps and extension, ligation, and indexing PCR steps followed by clean-up treatment with a solid-phase reversible immobilization method were performed according to the manufacturer’s instructions. The obtained DNA library was sequenced on an Illumina NovaSeq 6000 using a NovaSeq 6000 S4 Reagent Kit (Illumina, San Diego, United States) according to NovaSeq 6000 System User Guide Document #1000000019358 v02. After NGS, the quality of the raw sequence read data was determined by the Phred quality score at each cycle. A box plot containing the average quality at each cycle was created with FastQC version 0.11.5 (Andrews, 2010). A Phred quality score of 20 indicates 99% accuracy, and reads with a score > 20 are accepted as good quality. The adapter sequences were also trimmed from the raw sequence reads using Trim Galore v0.4.4_dev (Babraham Bioinformatics, 2017) and Cutadapt version 0.5.0 (Martin, 2011).

The cleaned reads were aligned to *A. thaliana* TAIR10 using BSMAP version 2.87 based on the Short Oligonucleotide Alignment Program (Xi and Li, 2009). The quality of the alignment data as a BAM file was assessed with Qualimap 2.2 (Okonechnikov et al., 2016). Only uniquely mapped reads were selected for sorting and indexing, and PCR duplicates were removed with SAMBAMBA version 0.5.9 (Tarasov et al., 2015). The methylation ratio of every single cytosine location was extracted from the mapping results using the methylation.py script in BSMAP. Methylation coverage for each cytosine in CG, CHH, and CHG was calculated. Each cytosine in CG, CHH, and CHG was annotated using the table browser function of the UCSC genome browser. Annotation included the functional location of the promoter region of each gene, which was defined as approximately 2 kb upstream of the transcription start site.

To estimate the bisulfite conversion rate, unmethylated lambda phage DNA (Cat# D1521; Promega) was added to the DNA prior to fragmentation. The bisulfite conversion rate was estimated to be 99.86–99.88% across the samples using lambda phage DNA.

### Bisulfite Sequence Analysis of the *DOF1.7* and *CBP1* Promoter Regions and Analysis of *DOF1.7* and *CBP1* Expression in *Arabidopsis thaliana* Ecotype Col-0 Transformed With 35S:2b.Ho-FLAG and Control Plants

For bisulfite sequence analysis of the *DOF1.7* and *CBP1* promoter regions, genomic DNA was isolated from the leaves of 35S:2b.Ho-transformed plants (Col:2b.Ho-FLAG) and vector-transformed control plants. Bisulfite conversion of genomic DNA was performed using an EpiTect Fast DNA Bisulfite Kit (Qiagen) according to the instruction manual and used as a template for PCR. Approximately 200–300 nucleotides of the promoter regions of *DOF1.7* and *CBP1* were amplified by PCR in a mixture of 1 × EpiTaq PCR buffer, 2.5 mM MgCl_2_, 0.3 mM each dNTP, 1.25 U TaKaRa EpiTaq HS, and 50 ng of template DNA with a set of 0.4 μM primers (DOF.Pro.AT1G51700.F2 and DOF.Pro.AT1G51700.R2 for the *DOF1.7* promoter; CBP.Pro.AT2G15890.F2 and CBP.Pro.AT2G15890.R2 for the *CBP1* promoter; Supplementary Table 5). PCR was performed for 30 cycles at 98°C for 10 s, 55°C for 30 s, and 72°C for 1 min. As a control, the genomic regions of *MEA-ISR* and *SUP* were amplified by the same procedure using a set of primers (MEA-ISR-BiF and MEA-ISR-BiR for the *MEA-ISR* fragment; SUP-BiF1 and SUP-BiR1 for the *SUP* fragment; Supplementary Table 5), because *MEA-ISR* is constitutively hypermethylated and *SUP* is constitutively hypomethylated (Sato et al., 2017). Deoxyriboadenosine was attached to the 3′-terminus of the PCR-amplified DNA and then cloned into the pTA2 vector using TArget Clone™-Plus (Toyobo, Osaka, Japan). Nucleotide sequences of 10 independent plasmid clones of each PCR-amplified DNA were determined by Sanger sequencing. The distribution and ratio of methylated cytosine residues were analyzed by Kismeth software (Gruntman et al., 2008).

### Analysis of *DOF1.7* and *CBP1* Expression in *Arabidopsis thaliana* Ecotype Col-0 Persistently Infected With CMV(Ho)tr- and Non-infected Plants

Relative levels of *DOF1.7* and *CBP1* transcripts were measured by qPCR. Total RNA was also isolated from main root tissues of corresponding CMV(Ho)tr- or mock-inoculated plants at 7 dpi. At the same time, total RNA was extracted at 7 dpi from CMV(Ho)tr- or mock-inoculated leaves of 4-week-old Col-0 plants, which were pre-treated with 150 mM NaCl for 24 h. qPCR for measurement of *DOF1.7* and *CBP1* transcripts was performed in triplicate 20-μl reactions containing 2 μl template cDNA, 0.4 mM DOF1.7- or CBP1-specific primers (Dof1-F and Dof1-R for *DOF1.7*; CBP1-F2 and CBP1-R2 for *CBP1*; Supplementary Table 6), 1 × ROX Reference Dye, and 1 × SYBR Premix Ex Taq II (Tli RNase H plus; Takara Bio) using the 7300 Real-Time PCR System (Applied Biosystems). PCR conditions and data analysis were performed as previously described (Takahashi et al., 2014). As an internal control, *A. thaliana UBQ5* transcript levels were measured by qPCR with the primers RTUBQ5-F1 and RTUBQ5-R1 (Supplementary Table 6). *DOF1.7* and *CBP1* mRNA levels were normalized relative to the values of the constitutively expressed *UBQ5* mRNA.

### Experimental Design and Statistical Analyses

For ELISA, total RNA extraction for northern blot and qPCR analysis, and western blot analysis, which were described in detail above, nine virus-inoculated *A. thaliana* plants and other nine plants mock-inoculated with 50 mM sodium phosphate buffer (pH 7.2) were used for each experiment. Three pieces of leaf tissues were randomly cut out from three inoculated leaves of each plant and were combined to make one sample. As a result, three biological replicates of virus- and mock-inoculated control plants, respectively, were set up for each experiment. In each experiment, the average ± standard deviation (SD) of the values of three independent test samples and three independent control samples was calculated. For statistical analysis, the comparison between two groups, virus-inoculated sample and mock-inoculated control sample, was subjected to Welch’s *t*-test, since a heteroscedasticity of two data variance was revealed by *F*-test. For multi-group comparison, the analysis was done by Turkey’s test. The same experiments were repeated three times independently for retrieving data of triple biological repetitions, and the representative result is shown.

For the evaluation of plant response to virus infection, symptom development, visualization of virus distribution in the infected plant by the tissue-printing method, and main and lateral root growth, at least three plants in one experiment were used. To analyze the influence of 2b protein on the morphological phenotype to *A. thaliana*, three independent transgenic lines expressing HA epitope sequence-tagged 2b transgene of CMV(Ho) or CMV(Y) were used for one experiment. All experiments were independently repeated three times for retrieving data of triple biological repetitions, and the representative result is shown.

For co-immunoprecipitation experiment, which was described in detail above, three independent transgenic lines expressing FLAG epitope sequence-tagged 2b transgene of CMV(Ho) and HA epitope sequence-tagged AGO cDNA clones under the control of CaMV 35S promoter were used for one experiment. To analyze the interaction between FLAG epitope sequence-tagged 2b protein of CMV(Ho) and HA epitope sequence-tagged AGO proteins by *Agrobacterium*-mediated transient gene expression method using *N. benthamiana*, the set of binary vector constructs encoding 2b protein and each AGO protein, respectively, were transiently co-expressed in three leaves of *N. benthamiana*, and three infiltrated leaves were combined to immunologically detect FLAG-2b and AGO proteins. All experiments were independently repeated three times for retrieving data of triple biological repetitions, and the representative result is shown.

To conduct whole-genome bisulfite sequencing (WGBS) analysis, three plants inoculated with CMV(Ho)tr and three mock-inoculated plants were used. Three pieces of leaf tissues were randomly cut out from CMV(Ho)tr- or mock-inoculated leaves of each plant. Leaf tissues collected from three inoculated plants (total nine pieces of leaf tissues) were combined to make one bulk sample, which was used for DNA extraction according to the procedure described above. In practice, the WGBS analysis using NGS cannot be done repeatedly. Thus, to confirm the reproducibility, we picked up two genes: *DOF1.7* and *CBP1*, whose promoter regions were hypomethylated according to the result obtained from the WGBS analysis, and the level of cytosine methylation in the *DOF1.7* and *CBP1* promoter regions between *A. thaliana* transformed with FLAG epitope sequence-tagged 2b gene of CMV(Ho) (35S:2b.Ho-FLAG) and vector control (pRI201-AN) was analyzed by bisulfite sequence analysis according to the procedure described above. The comparative analysis of the bisulfite sequence analysis of the *DOF1.7* and *CBP1* promoter regions was repeated three times for retrieving data of triple biological repetitions, and the representative result is shown.

## Results

### Characterization of CMV(Ho) Isolated From Virus-Infected *Arabidopsis halleri*

In a survey of naturally growing *A. halleri* in an area of an abandoned “Hosokura” mine in the Tohoku region of Japan (Supplementary Figures 5A–C), the CP of CMV was detected in varying levels by western blotting in 72 out of 103 symptomless plants derived from an independent plant community (Supplementary Figure 5D). No aphids were observed on any plant because the sampling was performed in early April, 2017, when the average of temperature at the sampled area was approximately 8°C. The 72 CMV-infected plants were not infected with tobacco mosaic virus, turnip mosaic virus, or cauliflower mosaic virus (CaMV), according to analysis using ImmunoStrip^®^ (Agdia, Elkhart, IN), designed for screening their CPs, or by western blotting (data not shown). The observations indicated that *A. halleri* plants in this area were infected with CMV without exhibiting typical symptoms.

**FIGURE 1:**
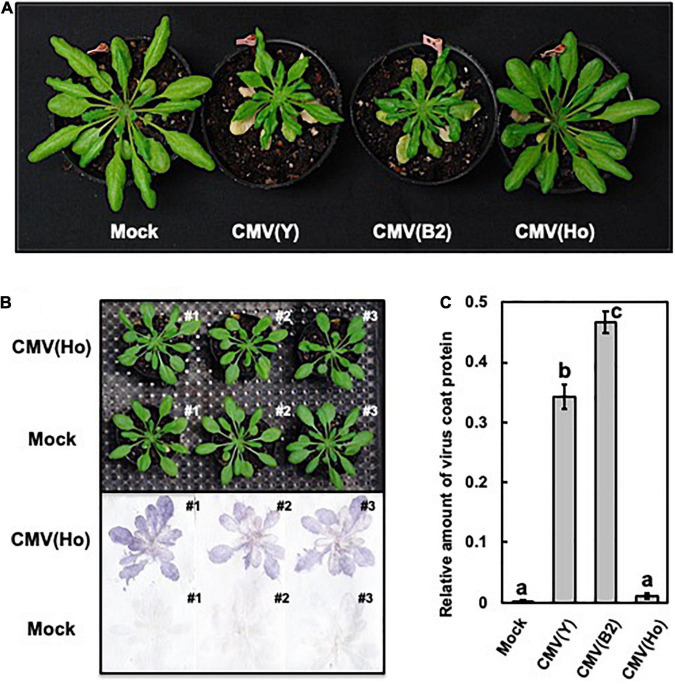
Observation of systemic symptoms and accumulation of virus coat protein in cucumber mosaic virus (CMV)-infected *A. thaliana* ecotype Col-0. Photographs of Col-0 plants at 21 days post-infection (dpi) with CMV(Ho) and two virulent strains of CMV [CMV(Y) and CMV(B2)]. As a control, Col-0 plants were rub-inoculated with 50 mM sodium phosphate buffer (pH 7.0) (mock) **(A)**. Virus coat protein in CMV(Ho)- and mock-inoculated plants at 21 dpi was detected by a tissue-printing method **(B)**. The amounts of coat protein in the inoculated leaves of three independent CMV(Ho)-, CMV(Y)-, or CMV(B2)-infected Col-0 plants and mock-inoculated plants were quantified by an enzyme-linked immunosorbent assay **(C)**. The average of the relative amounts of virus coat protein in virus- or mock-inoculated leaves (*n* = 3) is shown by a bar chart with error bars (*n* = 3, SD). The different letters indicate statistically significant differences in the average of relative amounts of the coat protein (Tukey’s test, *p* < 0.05).

To further investigate the CMV with which *A. halleri* was infected, a homogenous isolate, designated CMV(Ho), was purified *via* a local lesion host *C. amaranticolor* through five times of sequential passages. When fully expanded leaves of CMV-free *A. halleri* cv. AHG347 Winterclean were mechanically back sap-inoculated with the CMV(Ho) isolate, no symptoms developed on either virus-inoculated leaves or non-inoculated upper/systemic leaves (Supplementary Figure 6A). However, in the non-inoculated upper leaves of four out of the four CMV(Ho)-infected plants at 21 dpi, virus CP was clearly detected (Supplementary Figure 6B), indicating the occurrence of a systemic infection. On the other hand, in *A. halleri* cv. AHG347 Winterclean inoculated with virulent CMV strain [CMV(Y)], severe stunting symptoms developed (Supplementary Figure 6A), and the CP was also detected immunologically at a higher level of its accumulation in CMV(Ho) (Supplementary Figure 6B). Thus, *A. halleri* is essentially susceptible to CMV, but symptom development and accumulated level of the CP in *A. halleri* seem to be dependent on the infecting CMV strains or isolates. Likewise, when *A. thaliana* ecotype Col-0 was inoculated with CMV(Ho), no symptoms were observed at 21 dpi, but infection with CMV(Ho) was detected immunologically by a tissue blotting method (Figures 1A,B). When, as controls, Col-0 plants were infected with two virulent strains of CMV [CMV(Y) and CMV(B2)], clear systemic symptoms were induced (Figure 1A). Quantitative measurements of virus CP in CMV(Ho)-inoculated leaves revealed significantly less CP in CMV(Ho)-infected leaves compared to those infected with CMV(Y) or CMV(B2) (Figure 1C).

**FIGURE 2:**
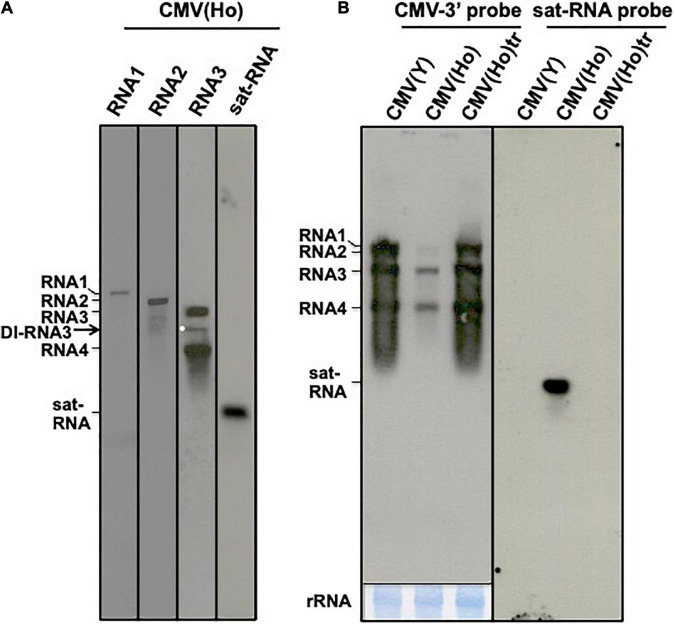
Northern hybridization analysis of CMV(Ho) RNAs. CMV(Ho) RNA, which was isolated from purified virion, was analyzed by northern hybridization using DIG-labeled probes specific to CMV RNA1, RNA2, RNA3, and satellite RNA (sat-RNA), respectively **(A)**. A white dot indicates a band of defective interfering (DI)-RNA3. Total RNA, which was isolated from CMV(Ho), CMV(Ho)tr, or virulent CMV(Y)-inoculated leaves of *A. thaliana* ecotype Col-0, was analyzed by northern hybridization using DIG-labeled probe (CMV-3′ probe) binding to 3′-conserved regions of CMV RNA1, RNA2, and RNA3 and DIG-labeled probe (sat-RNA probe) binding to CMV satellite RNA, respectively **(B)**. As an internal control, rRNA on the blotting membrane is shown at the lower panel by methylene blue staining. After hybridization followed by washing and incubation with CDP-Star substrate, the membrane was exposed for 1 min.

To further study the underlying mechanism of the symptomless and lowered infection rate of CMV(Ho) on *A. thaliana* and *A. halleri*, the genetic composition of the virus isolate was analyzed. To this end, viral RNA was purified from CMV(Ho) and detected by northern blot analysis using specific probes to CMV RNA1, RNA2, and RNA3 and CMV sat-RNA. As shown in Figure 2A, besides CMV(Ho) RNA1, RNA2, and RNA3, bands smaller in size than RNA3 were detected using the RNA3-specific probe (Figure 2A). Moreover, a ∼500-bp band was observed on northern blots using a probe for CMV sat-RNA (Figure 2A), suggesting that CMV(Ho) contains a sat-RNA molecule.

First-strand cDNA synthesis of RNA1, RNA2, RNA3, and sat-RNA of CMV(Ho) and subsequent PCR amplification, using primers specific to the 5′- and 3′-ends of each CMV RNA molecule, rendered DNA fragments of expected sizes corresponding to full-length RNA1, RNA2, RNA3, and sat-RNA (data not shown). In addition to the DNA fragment for RNA3, smaller RNA3-specific DNA molecules were detected (data not shown). This was interesting but not entirely unexpected, as in agreement with the band patterns of CMV(Ho) RNA3 observed during northern blot analysis (Figure 2A).

The DNA fragments corresponding to the full-length RNA1, RNA2, RNA3, and two short (most predominant) RNA3 molecules were cloned downstream of a T7 promoter in pUC18, and their nucleotide sequence was determined and registered in the GenBank/EMBL/DDBJ databases under the accession numbers LC593244 (RNA1), LC593245 (RNA2), and LC593246 (RNA3). Phylogenetic tree analysis of the deduced amino acid sequence of the CP of CMV(Ho) with those of other 11 CMV strains indicated that CMV(Ho) belongs to CMV subgroup IA (Supplementary Figure 7).

The nucleotide sequences of the two short RNA3 cDNA clones, designated CMV(Ho)RNA3.DI-1 and CMV(Ho)RNA3.DI-6, revealed a partial or complete deletion of the 3A protein-coding region and demonstrated their defective nature (Supplementary Figure 2). The nucleotide sequences of both DI-RNA3 cDNA clones were registered in the databases under the accession numbers LC593247 [CMV(Ho)RNA3.DI-1] and LC593248 [CMV(Ho)RNA3.DI-6]. Furthermore, when total RNA was used as a template for RT-PCR using primers designed to detect sat-RNAs, an approximately 500-bp DNA band corresponding to the size of a common sat-RNA was detected on agarose gel (data not shown). The nucleotide sequence of this sat-RNA cDNA was determined and deposited (accession number: LC593249). These results altogether indicated that CMV(Ho) consisted of a full set of CMV RNA genomic elements (RNA1, RNA2, and RNA3) and additionally at least two DI-RNA3 and sat-RNA.

### The Influence of DI-RNA3 and sat-RNA on CMV(Ho) Multiplication and Symptom Development

To investigate the infection resulting from a challenge with the full-length CMV RNA genomic elements only and the effect of the DI-RNA3 and sat-RNA on virus multiplication, the clones of CMV(Ho) RNA1, RNA2, RNA3, RNA3.DI-6, and sat-RNA were *in vitro* transcribed and subsequently inoculated onto *N. benthamiana* to propagate CMV(Ho)tr + RNA3.DI-6 and CMV(Ho)tr + sat-RNA, respectively (Supplementary Table 1 and Supplementary Figure 8).

When fully expanded leaves of *A. thaliana* ecotype Col-0 (35 days after sowing) were inoculated with CMV(Ho)tr, CMV(Ho)tr + RNA3.DI-6, or CMV(Ho)tr + sat-RNA, no symptoms developed at 14 dpi (data not shown). When infected leaves were sampled and analyzed by western blotting for the CP production, the leaves co-infected with CMV(Ho)tr + RNA3.DI-6 or CMV(Ho)tr + sat-RNA showed lower amounts than the leaves infected with only CMV(Ho)tr (Supplementary Figure 8A). Moreover, in quantitative measurement of CMV genomic RNA3 by qPCR, co-infection of RNA3.DI-6 or sat-RNA with CMV(Ho)tr reduced the amount of the genomic RNA3 in Col-0 leaves (Supplementary Figure 8B). The presence of the co-infection of RNA3.DI-6 or sat-RNA with CMV(Ho)tr was confirmed by RT-PCR (Supplementary Figure 8C).

The lower amounts of CMV RNA1, RNA2, and RNA3 accumulation in CMV(Ho)-inoculated Col-0 leaves as compared to CMV(Ho)tr- or CMV(Y)-inoculated leaves were also observed by northern blot analysis (Figure 2B), while the amount of CMV RNAs in CMV(Ho)tr-inoculated Col-0 leaves highly accumulated at similar levels in virulent CMV(Y)-inoculated Col-0 (Figure 2B). In addition, sat-RNA [present in CMV(Ho)] was detected in CMV(Ho)-inoculated leaves (Figure 2A), like the DI-RNA3.

**FIGURE 3:**
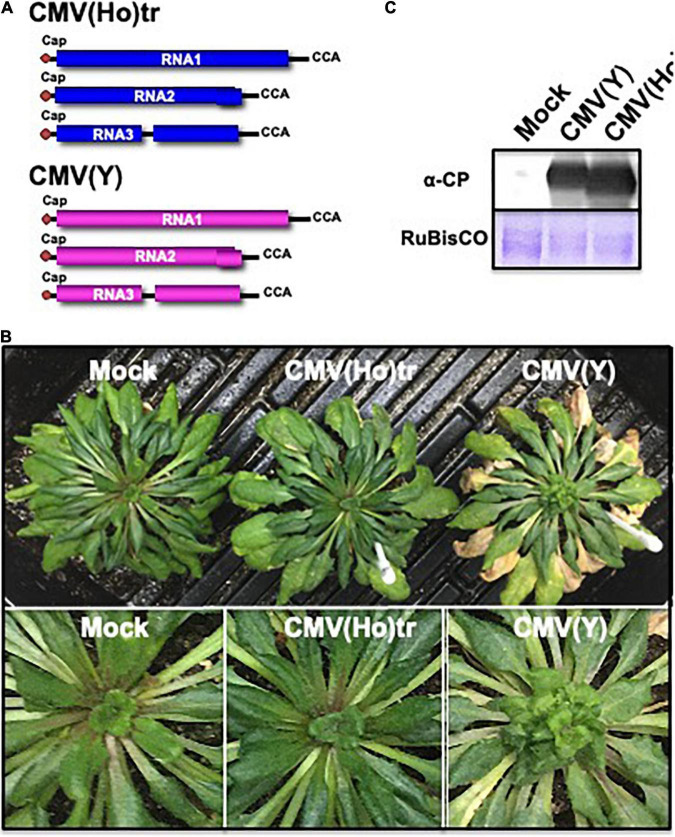
The response of *A. thaliana* ecotype Col-0 to CMV(Ho)tr and virulent CMV(Y) and detection of virus coat protein in virus-inoculated leaves. Schematic structures of *in vitro*-transcribed RNA1, RNA2, and RNA3 of CMV(Ho)tr and CMV(Y) are shown **(A)**. The 5′-end of each RNA has a ^7^mGpppG-cap structure (Cap), and the 3′-end of each RNA has the same nucleotide sequence (5′-CCA-3′). At 14 dpi, CMV(Ho) tr-, CMV(Y)-, and mock-inoculated plants were photographed **(B)**. Enlarged photographs of non-fully expanded young leaves of each plant are shown in the lower panel. Virus coat protein in CMV(Ho) tr-, CMV(Y)-, or mock-inoculated leaves was detected immunologically at 14 dpi. As an internal control, the band of ribulose-1,5-bisphosphate carboxylase/oxygenase (RuBisCO), which was detected by Coomassie brilliant blue (CBB) staining, is shown in **(C)**.

When fully expanded leaves of Col-0 were inoculated with CMV(Ho)tr next to virulent CMV(Y) (Figure 3A), CMV(Ho)tr-infected Col-0 plants did not show any symptoms, whereas CMV(Y)-infected plants developed a clear yellowing and some stunting at 14 dpi (Figure 3B). When leaf samples were analyzed for the presence of the CP, the amounts detected for CMV(Ho)tr-inoculated leaves were quite abundant and equal to those in CMV(Y)-inoculated leaves (Figure 3C). Thus, the accumulation of CMV RNA (Figure 2B) and CP in CMV(Ho)tr- and CMV(Y)-inoculated leaves indicates more or less similar infection rates for both viruses in Col-0 plants. Same infection rates of CMV(Ho)tr and virulent CMV(Y) were also observed in *A. halleri* (Supplementary Figure 6B), while severe systemic symptoms developed only in CMV(Y)-infected *A. halleri* (Supplementary Figure 6A). Therefore, although RNA3.DI-6 and sat-RNA of CMV(Ho) reduced CMV(Ho) multiplication, the asymptomatic phenotype in CMV(Ho)-infected *A. thaliana* and *A. halleri* is not dependent on the co-infection of RNA3.DI-6 or sat-RNA with CMV(Ho)tr.

### The Phenotype of *Arabidopsis thaliana* Infected With CMV(Ho)tr in Comparison With Non-infected Plants

**FIGURE 4:**
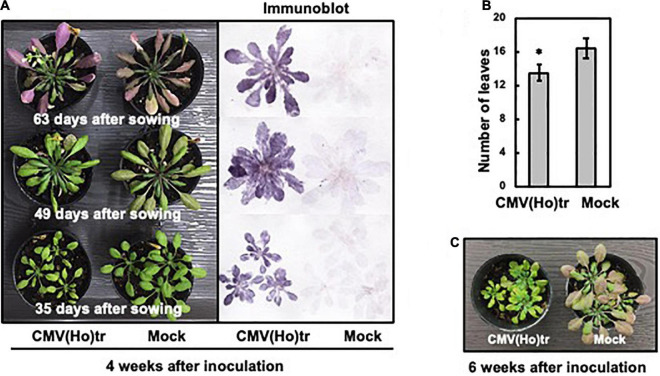
The influence of infection with CMV(Ho)tr on the growth of *A. thaliana* ecotype Col-0, which was assessed at three different growth stages. Fully expanded leaves of Col-0 plants, which were cultivated for 7, 21, or 35 days, were inoculated with CMV(Ho)tr or sodium phosphate buffer (pH 7.0) as a control (mock) and further cultivated for 4 weeks after inoculation [left panel in **(A)**]. Thus, the ages of each plant in **(A)** were different at 35, 49, and 63 days, respectively, but all at 4 weeks post-infection. After photographing the plants, virus coat protein was detected immunologically by a tissue-printing method [right panel in **(A)**]. The average number of detached leaves from three independent 35-day-old plants is shown in the bar chart with error bars (*n* = 10, SD). An asterisk denotes the statistically significant difference between CMV(Ho) and mock control plants (Welch’s *t*-test, *p* < 0.01) **(B)**. CMV(Ho)tr- and mock-inoculated plants, which were inoculated at 7 days after sowing and further cultivated for 6 weeks after inoculation [infected with CMV(Ho)tr at 7 days post-sowing], were photographed **(C)**.

To investigate the influence of CMV(Ho)tr infection on plants at three different growth stages, fully expanded leaves of Col-0 plants were inoculated with CMV(Ho)tr at 7, 21, or 35 days after sowing (data not shown) and monitored 4 weeks post-infection. Plants, which were 49 and 63 days old and challenged at 21 and 35 days post-sowing, respectively, did not show significant differences in their growth compared to healthy control plants (Figure 4A). On the other hand, and interestingly, the growth of 35-day-old plants infected with CMV(Ho)tr at 7 days post-sowing (early in development) was clearly delayed in comparison with control plants (Figure 4A). Although the leaves were as green as those of mock-inoculated (control) plants (Figure 4A), their number and size were clearly reduced (Figure 4B). Furthermore, at 6 weeks post-inoculation (49 days after sowing), the leaves of the control plants became red, possibly due to the accumulation of anthocyanins, which seemed to be indicative of leaf senescence, whereas those from CMV(Ho)tr-infected plants remained green and seemed to be in the nutrient growth period (Figure 4C). Systemic infection in all CMV(Ho)tr-inoculated plants was confirmed by immunological analysis of leaf tissue prints (Figure 4A). Altogether, these results indicated that CMV(Ho)tr infection did not seem to have strong negative effects on plant physiology and delayed the development and senescence.

**FIGURE 5:**
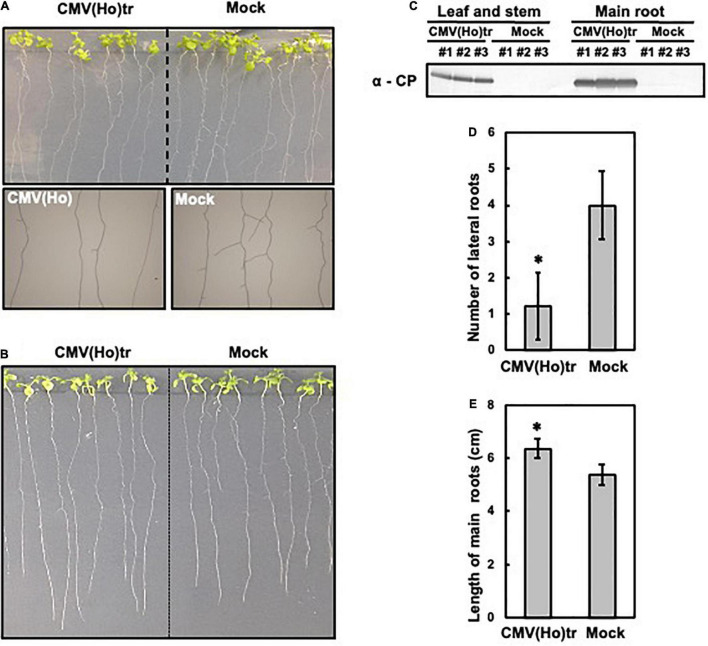
The influence of infection with CMV(Ho)tr on root growth of *A. thaliana* ecotype Col-0. Fully expanded leaves of Col-0 plants at 7 days after sowing on 1/2 Murashige–Skoog medium were inoculated with CMV(Ho)tr or sodium phosphate buffer (pH 7.0) (mock) as a control. Photographs of the development of lateral roots of CMV(Ho)tr- and mock-inoculated plants are shown in upper side in **(A)**, and enlarged photographs of the lateral roots are shown in the lower side in **(A)**. Photographs of the development of main root of CMV(Ho)tr- and mock-inoculated plants are shown **(B)**. Systemic infection with CMV(Ho)tr was confirmed by immunological detection of the coat protein in root tissue **(C)**. The number of lateral roots **(D)** and length of the main root **(E)** of independent CMV(Ho)tr- or mock-inoculated plants at 7 dpi are shown in the bar chart with error bars (*n* = 10, SD). An asterisk denotes the significant difference between CMV(Ho) and mock control plants (Welch’s *t*-test, *p* < 0.01).

To analyze the effect of CMV(Ho)tr infection on root growth and development, 7-day-old Col-0 seedlings were inoculated with CMV(Ho)tr, and the roots were analyzed at 7 dpi in comparison to non-inoculated plants (Figures 5A,B). The systemic spread of virus in main root and leaf tissues was confirmed by the detection of CP by western blotting (Figure 5C). While the development of lateral roots was suppressed in CMV(Ho)tr-infected plants in comparison with control plants (Figures 5A,D), the growth of the main root, in contrast, was enhanced in CMV(Ho)tr-infected plants (Figures 5B,E).

### Viral Determinant for Asymptomatic Phenotype in CMV(Ho)tr-Infected *Arabidopsis thaliana*

**FIGURE 6:**
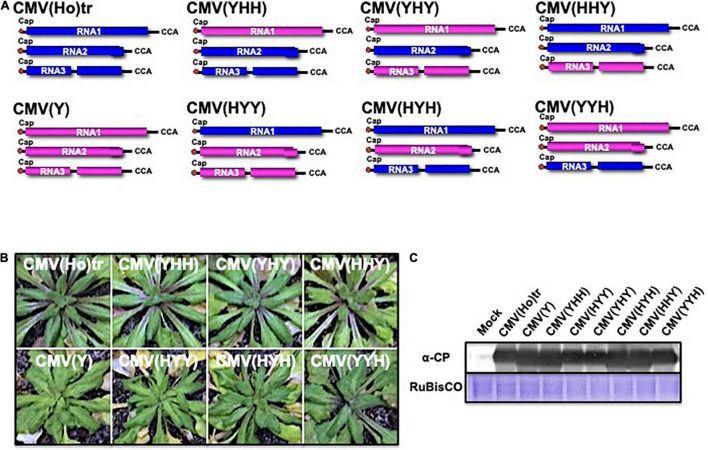
The response of *A. thaliana* ecotype Col-0 to infection with reassortant CMVs. Fully expanded leaves of Col-0 plants were inoculated with a series of CMV reassortants from the parental strains CMV(Ho)tr (RNA segments colored blue) and virulent CMV(Y) (RNA segments colored red): CMV(YHH), CMV(YHY), CMV(HHY), CMV(HYY), CMV(HYH), or CMV(YYH), in which the position and letter refer to the RNA segment (1, 2, or 3) and origin of the parental strains, respectively **(A)**. The 5′-end of each RNA has a ^7^mGpppG-cap structure (Cap), and the 3′-end of each RNA has the same nucleotide sequence (5′-CCA-3′). Col-0 plants were inoculated with CMV(Ho)tr or virulent CMV(Y) as controls. Photographs of young leaves of Col-0 plants infected with a series of reassortant CMVs, CMV(Ho)tr, or virulent CMV(Y) at 14 dpi are shown **(B)**. CMV coat protein in virus-infected leaves was detected immunologically by western blotting **(C)**. As an internal control, RuBisCO was detected by CBB staining and shown in the lower panel.

To identify the viral determinant for symptomatology in infection of *A. thaliana* ecotype Col-0 with CMV(Ho)tr, a series of reassortant CMVs between CMV(Ho)tr and virulent CMV(Y) were created by the combination of *in vitro* transcripts of RNA1, RNA2, and RNA3 between both CMV strains (Figure 6A). Reassortants containing CMV(Ho)tr RNA2, namely, CMV(YHH), CMV(YHY), and CMV(HHY), did not induce any symptoms, similar to CMV(Ho)tr infection, while all reassortants containing CMV(Y) RNA2, namely, CMV(HYY), CMV(HYH), and CMV(YYH), induced systemic symptoms at 14 dpi, which were clearly observed in non-fully expanded young leaves and similar to those of CMV(Y) (enlarged photograph of non-fully expanded young leaves in Figure 6B). Given that the production of the CP was similar among the plants infected with all reassortants (Figure 6C), the absence of disease symptoms during CMV(Ho)tr infection seemed to be correlated to CMV(Ho) RNA2, which encodes two proteins (2a and 2b) that function as RNA-dependent RNA polymerase and RNA-silencing suppressor, respectively.

**FIGURE 7:**
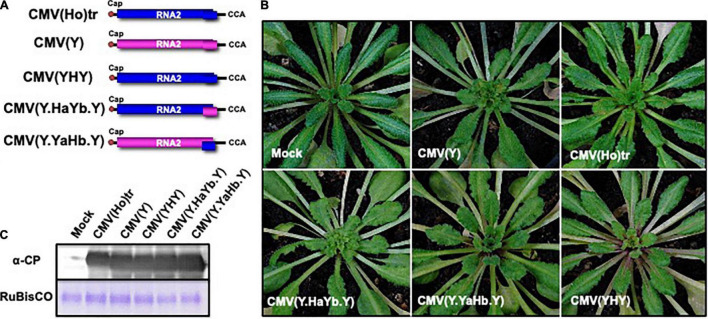
The response of *A. thaliana* ecotype Col-0 to infection with CMV containing chimeric RNA2 between CMV(Ho)tr and virulent CMV(Y). Fully expanded leaves of Col-0 plants were inoculated with CMV(Y.HaYb.Y) or CMV(Y.YaHb.Y) containing CMV(Y) RNA1 and RNA3 and chimeric RNA2 in which the two open reading frames (ORFs) encoding 2a and 2b proteins were reciprocally exchanged between CMV(Ho)tr and virulent CMV(Y). As a control, Col-0 plants were inoculated with CMV(Ho)tr, CMV(YHY), or virulent CMV(Y), which are shown in Figure 6A. The schematic structures of each CMV RNA2 are shown in **(A)**. The 5′-end of each RNA has a ^7^mGpppG-cap structure (Cap), and the 3′-end of each RNA has the same nucleotide sequence (5′-CCA-3′). Photographs of young leaves of Col-0 plants infected with CMV(Y.HaYb.Y), CMV(Y.YaHb.Y), CMV(Ho)tr, CMV(YHY), or CMV(Y) at 14 dpi are shown **(B)**. CMV coat protein in virus-infected leaves was detected immunologically by western blotting **(C)**.

When Col-0 plants were inoculated with CMV containing chimeric RNA2 in which the *2a* and *2b* genes were reciprocally exchanged between CMV(Ho)tr and CMV(Y), designated as CMV(Y.HaYb.Y) and CMV(Y.YaHb.Y) (Figure 7A), systemic symptoms developed at 14 dpi on non-fully expanded young leaves in Col-0 infected with CMV(Y.HaYb.Y) as well as CMV(Y), but not in Col-0 infected with CMV(Y.YaHb.Y) (Figure 7B). Despite differences in symptomatology between certain reassortants, the production levels of the CP were similar in all infected plants, irrespective of the reassortants (Figure 7C). These results indicated that the absence of disease symptoms is correlated to the *2b* gene of CMV(Ho)tr.

### Comparison of the RNA-Silencing Suppressor Activity of 2b Protein of CMV(Ho) and CMV(Y)

**FIGURE 8:**
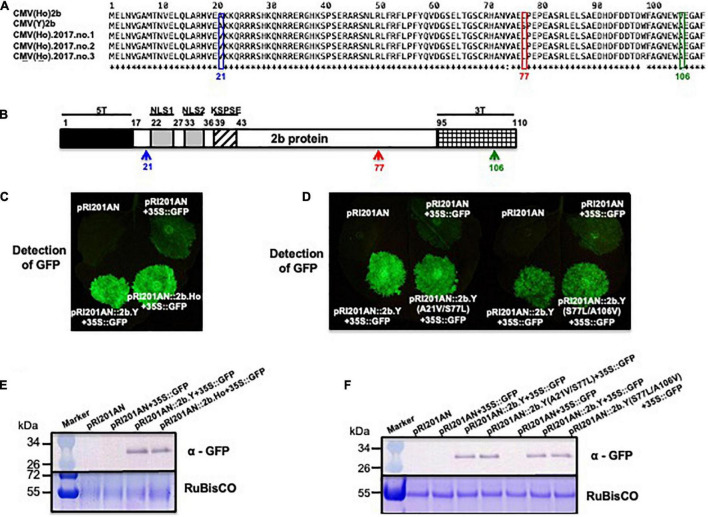
The analysis of the RNA-silencing suppressor activity of 2b proteins of CMV(Ho) and virulent CMV(Y) and 2b proteins of CMV(Y) carrying amino acid substitutions using *N. benthamiana* 16c. Alignment of the amino acid sequences of 2b protein encoded by RNA2 of CMV(Ho), CMV(Ho).2017.no.1, CMV(Ho).2017.no.2, CMV(Ho).2017.no.3, and virulent CMV(Y) **(A)**. Single amino acid substitution at position 77 in 2b protein from serine (S) in CMV(Y) to leucine (L) in CMV(Ho), CMV(Ho).2017.no.1, CMV(Ho).2017.no.2, and CMV(Ho).2017.no.3 is indicated by a red box. Single amino acid substitutions at position 21 in 2b protein from alanine (A) in virulent CMV(Y) to valine (V) in CMV(Ho).2017.no.1, CMV(Ho).2017.no.2, and CMV(Ho).2017.no.3 and at amino acid position 106 in 2b protein from alanine (A) in virulent CMV(Y) to valine (V) in CMV(Ho) are shown by blue and green boxes, respectively. In the schematic view of 2b protein **(B)**, the functional domains of 2b protein for nuclear localization (NLS1 and NLS2) and phosphorylation (KSPSE), which are associated with RNA-silencing suppressor activity, are shown by gray and striped pattern boxes, respectively. T5 and 3T indicate the variable regions at the N- and C-terminals of 2b protein, respectively. pRI201AN:2b.Ho, pRI201AN:2b.Y, or pRI201AN were transiently co-expressed with 35S:GFP in the leaves of *N. benthamiana* 16c by the agroinfiltration method **(C)**. pRI201AN:2b.Y, pRI201AN:2b.Y(S77L/A106V), pRI201AN:2b.Y(A21V/S77L), pRI201AN:2b.Y, pRI201AN:2b.Ho, or pRI201AN were transiently co-expressed with 35S:GFP in the leaves of *N. benthamiana* 16c **(D)**. As a control, pRI201AN was only agroinfiltrated **(C,D)**. The GFP signal was visualized under a dark field **(C,D)**. The accumulation of GFP was detected immunologically using an antibody against GFP **(E,F)**. As an internal control, RuBisCO was detected by CBB staining and shown in the lower panel.

Amino acid (aa) sequence comparison of the 2b protein from CMV(Ho)tr and CMV(Y) indicated the presence of two aa substitutions, namely, Ser (S) to Leu (L) at aa position 77 (S77L) and Ala (A) to Val (V) at aa position 106 (A106V) (Figure 8A). Furthermore, the aa sequences of the *2b* protein gene from three independent CMV isolates collected from independent communities of *A. halleri* from the same geographical region [i.e., CMV(Ho_2017.no.1), CMV(Ho_2017.no.2), and CMV(Ho_2017.no.3)] also indicated another variant, i.e., besides the S77L, instead of A106V, a substitution of A to V at aa position 21 (A21V) was observed (Figure 8A). The observed aa substitutions in 2b protein (S77L/A106V and A21V/S77L) were both located outside of domains needed for RNA-silencing suppressor activity (Lewsey et al., 2010a; Figure 8B).

To test and confirm the RNA-silencing suppressor activity of 2b from CMV(Ho) and CMV(Y), the leaves of *GFP*-transformed *N. benthamiana* 16c (Haseloff et al., 1997; Ruiz et al., 1998) were agroinfiltrated (Figure 8C) with a combination of pRI201AN:GFP (binary GFP-expressor construct) and a binary expressor construct of pRI201AN:2b.Y (binary 2b.Y-expressor construct) or pRI201AN:2b.Ho (binary 2b.Ho-expressor construct), respectively (Figures 8C,E and Supplementary Figures 4A, 9). As controls, the leaves were co-infiltrated with pRI201AN:GFP or empty vector pRI201AN (Figures 8C,E and Supplementary Figures 4A, 9). While transient *GFP* expression from pRI201AN:GFP became silenced in 16c leaves during co-infiltration with the negative control pRI201AN, its silencing was suppressed with pRI201AN:2b.Y or pRI201AN:2b.Ho (Figures 8C,E), indicating a functional 2b of CMV(Ho). The quantitative level of suppression, as measured by the amount of GFP fluorescence, was similar in pRI201AN:2b.Y and pRI201AN:2b.Ho (Supplementary Figure 9A). Western immunoblot analyses on samples collected from the agroinfiltrated leaves showed no difference in the accumulation of HA epitope-tagged 2b proteins encoded by pRI201AN:2b.Y and pRI201AN:2b.Ho (Supplementary Figure 10A).

To analyze the influence of the aa substitutions observed in 2b.Ho on RNA-silencing suppressor activity of 2b, the leaves of *GFP*-transformed *N. benthamiana* 16c were agroinfiltrated with a combination of pRI201AN:GFP (binary GFP-expresser construct) and a binary expressor construct of mutant 2b.Y containing either of the two observed 2b.Ho aa substitutions (Figures 8D,F and Supplementary Figures 4A, 9). While transient *GFP* expression from pRI201AN:GFP became silenced in 16c leaves during co-infiltration with the negative control pRI201AN, its silencing was suppressed with pRI201AN:2b.Y(S77L/A106V) or pRI201AN:2b.Y(A21V/S77L) (Figures 8D,F). The quantitative level of suppression, as measured by the amount of GFP fluorescence, was similar with that from the positive control pRI201AN:2b.Y (Supplementary Figure 9B). Western immunoblot analyses on samples collected from the agroinfiltrated leaves showed no difference in the accumulation of HA epitope-tagged 2b proteins encoded by pRI201AN:2b.Y(S77L/A106V), pRI201AN:2b.Y(A21V/S77L), and pRI201AN:2b.Y (Supplementary Figure 10B). These results indicated that the two aa substitutions observed in 2b.Ho did not affect the RNA-silencing suppressor activity of CMV(Y) 2b protein.

### The Role of 2b Protein on Asymptomatic Infection of *Arabidopsis thaliana* With CMV(Ho)

**FIGURE 9:**
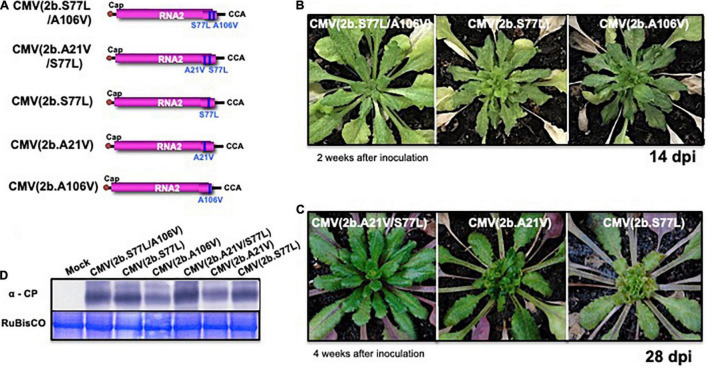
The response of *A. thaliana* ecotype Col-0 to infection with CMV carrying single-nucleotide substitutions in the *2b* gene of RNA2. The schematic structures of each CMV(Y) RNA2 encoding 2b protein with the single amino acid substitutions A21V, S77L, or A106V and their combinations (A21V/S77L or S77L/A106V), respectively, are shown in **(A)**. CMV(2b.S77L/A106V), CMV(2b.A21V/S77L), CMV(2b.S77L), CMV(2b.A21V), and CMV(2b.A106V) contain CMV(Y) RNA1 and RNA3 and those CMV(Y) RNA2 shown in **(A)**. The 5′-end of each RNA has a ^7^mGpppG-cap structure (Cap), and the 3′-end of each RNA has the same nucleotide sequence (5′-CCA-3′). Fully expanded leaves of Col-0 were inoculated with CMV(2b.S77L/A106V), CMV(2b.A21V/S77L), CMV(2b.S77L), CMV(2b.A21V), or CMV(2b.A106V). Photographs of young leaves of Col-0 infected at 14 and 28 dpi are shown in **(B,C)**. CMV coat protein in virus-infected leaves was detected immunologically by western blotting **(D)**. As an internal control, RuBisCO was detected by CBB staining and shown in the lower panel.

When Col-0 plants were inoculated with each of the following virus: CMV(2b.A21V), CMV(2b.S77L), CMV(2b.A106V), CMV(2b.S77L/A106V), and CMV(2b.A21V/S77L), respectively (Figure 9A), no symptoms appeared in those infected with a double substitution, CMV(2b.S77L/A106V) or CMV(2b.A21V/S77L) (Figures 9B,C). In contrast, severe stunting symptoms developed in the plants infected with CMV(2b.S77L), CMV(2b.A21V), or CMV(2b.A106V) (Figures 9B,C). Upon western immunoblot analysis of systemic (non-inoculated) leaves, similar production levels of the CP were observed with all mutant 2b constructs (Figure 9D).

**FIGURE 10:**
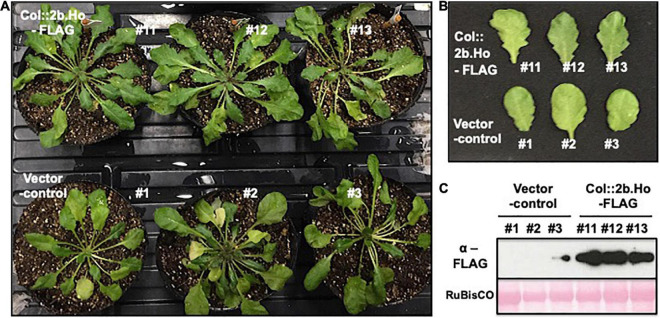
Asymptomatic phenotype of *A. thaliana* ecotype Col-0 transformed with FLAG epitope sequence-tagged cDNA for *2b* RNA of CMV(Ho). Three independent lines of Col-0 plants transformed with FLAG epitope sequence-tagged cDNA for *2b* RNA of CMV(Ho) (Col:2b.Ho-FLAG #11, #12, and #13) and three independent lines of pRI201AN-transformed plants (vector control #1, #2, and #3), as the control, were photographed at 35 days after sowing **(A)**. Fully expanded leaves of the transformed and control plants were photographed **(B)**. FLAG-tagged 2b protein was detected immunologically by western blotting **(C)**. As an internal control, RuBisCO was detected by Ponceau-S staining and shown in the lower panel.

To investigate whether the expression of 2b protein was sufficient to cause a symptom-like phenotype independently from the other CMV proteins, the FLAG-epitope-tagged *2b* gene of CMV(Ho) or CMV(Y) (p35S:2b.Ho-FLAG or p35S:2b.Y-FLAG in Supplementary Figure 4B) was transformed into Col-0 plants (Figure 10 and Supplementary Figure 11). An analysis of three (randomly selected) independent F3 transgenic lines carrying homozygous *2b.Ho-FLAG* and accumulating FLAG epitope-tagged 2b.Ho proteins (#11, #12, and #13) revealed a similar growth as three independent non-transgenic control plants (#1, #2, and #3) at 35 days after sowing (Figures 10A–C). Three months after sowing, still no significant differences in growth were observed between the *2b.Ho-FLAG*-expressing transformants and control plants, while Col-0 plants transformed with *2b.Y-FLAG* exhibited the symptom-like phenotype (Supplementary Figures 11A,B). These results indicated that 2b presents the major determinant of disease symptomatology, in which aa substitutions of S77L with either A21V or A106V change the symptomatic infection [CMV(Y)] into a symptomless one [CMV(Ho)].

### The Interaction of CMV(Ho) 2b Protein With AGO Proteins

**FIGURE 11:**
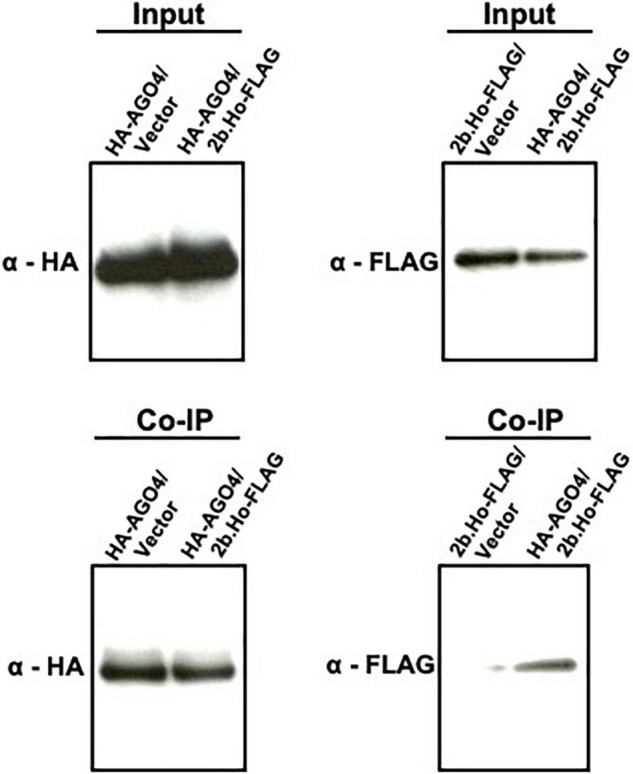
Co-immunoprecipitation (Co-IP) analysis of AGO4 protein with 2b protein of CMV(Ho) employing the transient expression agroinfiltration method in *N. benthamiana*. Hemagglutinin (HA) epitope sequence-tagged cDNA for *AGO4* RNA (p35S:HA-AGO4 in Supplementary Figure 4B) and FLAG epitope sequence-tagged cDNA for *2b* RNA of CMV(Ho) (p35S:2b.Ho-FLAG in Supplementary Figure 4B) were transiently expressed by the agroinfiltration method in *N. benthamiana* leaves (HA-AGO4/2b.Ho-FLAG). As a control, HA epitope sequence-tagged cDNA for *AGO4* RNA (p35S:HA-AGO4 in Supplementary Figure 4B) or FLAG epitope sequence-tagged cDNA for *2b* RNA of CMV(Ho) (p35S:2b.Ho-FLAG in Supplementary Figure 4B) was also transiently expressed (HA-AGO4/vector and 2b-FLAG/vector, respectively). HA-tagged AGO4 protein (HA-AGO4) and FLAG-tagged 2b protein (2b-FLAG) in leaf homogenates from agroinfiltrated *N. benthamiana* leaves, which were used as “input” for Co-IP analysis, were detected immunologically using antibodies against the HA (α-HA) and FLAG (α-FLAG) epitopes, respectively. In Co-IP, HA-AGO4 binding to anti-HA agarose beads was detected immunologically by α-HA, and 2b-FLAG, which was co-immunoprecipitated with HA-AGO4, was assessed using α-FLAG.

While HA-AGO6, HA-AGO7, HA-AGO8, HA-AGO9, and HA-AGO10 proteins were not reproducibly detected after agroinfiltration of their encoding constructs in *N. benthamiana* leaves (data not shown), HA-AGO1, HA-AGO2, HA-AGO3, HA-AGO4, and HA-AGO5 proteins accumulated in the infiltrated leaves (Supplementary Figure 12). Upon co-expression of the latter with 2b.Ho-FLAG and subsequent Co-IP analysis, the 2b.Ho-FLAG protein was co-immunoprecipitated with HA-AGO4 and HA-AGO5, but not with HA-AGO1, HA-AGO2, or HA-AGO3 (Supplementary Figure 12). A Co-IP on the input samples again showed that AGO4 co-immunoprecipitated with 2b (Figure 11), confirming the earlier observations on AGO4 and CMV(Ho) 2b interaction.

To further substantiate these findings, the interaction of AGO4 with CMV(Ho) 2b was also examined using transgenic plants constitutively co-expressing AGO4 and 2b of CMV(Ho): Col:35Spro.2b.Ho-FLAG/35Spro.HA-AGO4 (lines #1, #2, and #3) in Supplementary Figure 13. Prior to Co-IP analysis, the expression of both CMV(Ho) 2b-FLAG and HA-AGO4 was confirmed in the plants carrying 35S:HA-AGO4, 35S:2b.Ho-FLAG, or 35Spro.2b.Ho-FLAG/35Spro.HA-AGO4 (Supplementary Figures 13A,B). Upon Co-IP analysis on leaf homogenates of these plants, 2b-FLAG was observed to co-immunoprecipitate with HA-AGO4 protein (Supplementary Figures 13C,D), suggesting that the 2b protein of CMV(Ho) seems to interact to AGO4 protein in plants showing no symptoms.

### Comparative Analysis of Cytosine Hypomethylation in the Whole Genome in CMV(Ho)tr- and Mock-Inoculated *Arabidopsis thaliana* Plants

Given that AGO4 is reported to be associated with the regulation of cytosine methylation levels on the genome of *A. thaliana* (Law and Jacobsen, 2010), cytosine hypomethylation levels in the whole genome of *A. thaliana* ecotype Col-0 leaves inoculated with CMV(Ho)tr were compared with those in control Col-0 plants by whole-genome bisulfite sequencing analysis. In comparison with cytosine hypomethylation levels in the chromosomes of mock-inoculated plants (22.2% in CG, 8.9% in CHG, and 3.0% in CHH), cytosine methylation in CMV(Ho)tr-inoculated plants decreased to 20.8% in CG, 7.9% in CHG, and 2.3% in CHH (Table 1 and Supplementary Figure 14).

**TABLE 1:** Methylation coverage and ratio of every single cytosine in CG, CHG, and CHH.

Sequence context	Sample	Total coverage	Methylated coverage	% Methylation^a^
CG	CMV(Ho)tr	154,623,328	32,098,859	20.8
CG	Mock	133,539,058	29,677,092	22.2
CHG	CMV(Ho)tr	153,836,755	12,153,913	7.9
CHG	Mock	131,113,478	11,659,672	8.9
CHH	CMV(Ho)tr	737,490,592	16,900,124	2.3
CHH	Mock	608,528,950	18,219,559	3.0

*^a^Methylation ratio of every single cytosine higher than 1 CT was called using the methyratio.py script in BSMAP.*

Thus, CMV(Ho)tr infection seemed to induce cytosine hypomethylation of CG, CHG, and CHH. Further analysis revealed the occurrence of hypomethylation in 82 promoter regions (∼2 kb) (Supplementary Figure 15 and Supplementary Table 7).

**FIGURE 12:**
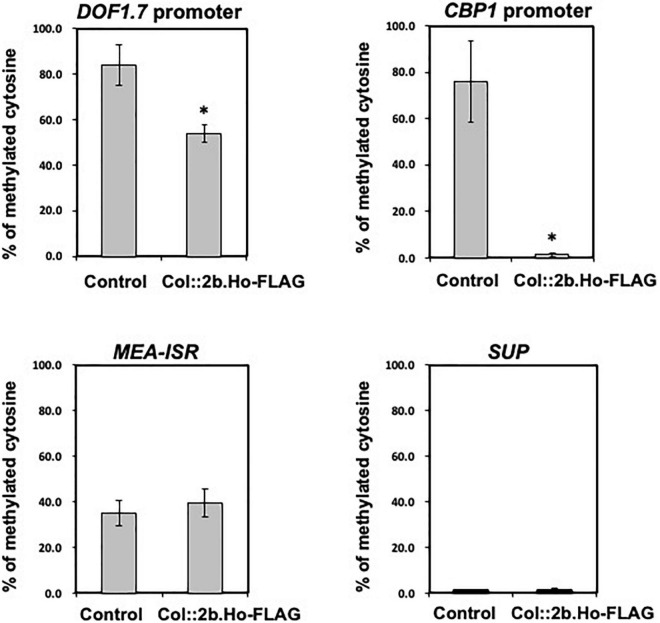
Levels of hypomethylated cytosine in the promoter regions of two genes encoding the transcriptional regulators DOF1.7 and CBP1 by bisulfite sequencing analysis in CMV(Ho)tr- and mock-inoculated leaves of *A. thaliana* ecotype Col-0. Percentages of methylated cytosine of CG, CHG, and CHH in the promoter regions of *DOF1.7* and *CBP1* in three independent CMV(Ho) *2b*-transformed Col-0 (Col:35Spro.2b.Ho-FLAG) and vector-transformed (control) Col-0 plants are shown in the bar chart with error bars (*n* = 3, SD). As a control, the percentages of methylated cytosine of those transformants in the genomic regions of *MEA-ISR*, which is constitutively hypermethylated, and *SUP*, which is constitutively hypomethylated, are also shown by a bar chart with error bars (*n* = 3, SD). Asterisks denote significant differences between Col:2b.Ho-FLAG and control (Welch’s *t*-test, *p* < 0.05).

To determine whether 2b protein induced cytosine hypomethylation of the Col-0 genome, cytosine methylation of the upstream promoter regions of two of those 82 corresponding genes {AGI codes At2g15890 and At1g51700, encoding transcriptional regulators [central cell guidance (CCG)-binding protein 1 (CBP1), also known as MEE14, and Dof1.7 transcription factor (DOF1.7)]} was analyzed from the genomes of Col:2b.Ho-FLAG and vector control plants by bisulfite sequencing (Figure 12). The methylation levels in the ∼300-bp promoter region upstream of *CBP1* and *DOF1.7* were both significantly decreased in Col:2b.Ho-FLAG plants in comparison with control plants (Figure 12 and Supplementary Figures 16, 17), which is similar to the situation as observed with CMV(Ho)tr-infected plants. There was no difference in cytosine methylation in *MEA-ISR*, which is constitutively hypermethylated, or *SUP*, which is constitutively hypomethylated, between Col:2b.Ho-FLAG plants and vector control plants (Figure 12). The nucleotide positions of cytosine hypomethylation in the promoter regions of *CBP1* and *DOF1.7* in Col:2b.Ho-FLAG, but hypermethylated in vector control plants, are shown in Supplementary Figures 16, 17, respectively. These results altogether indicated that cytosine hypomethylation of CG, CHG, and CHH in the promoter regions of *CBP1* and *DOF1.7* is correlated with the accumulation of 2b protein encoded by CMV(Ho) RNA2.

### Modulation of *CBP1* and *DOF1.7* Expression by Cytosine Hypomethylation of Their Promoter Regions

**FIGURE 13:**
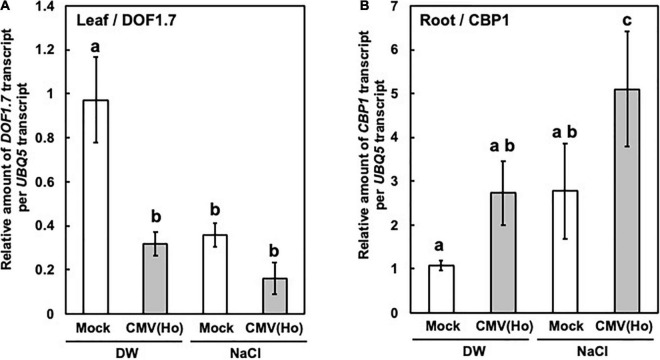
*DOF1.7* and *CBP1* expression in CMV(Ho)tr-infected and mock-infected *A. thaliana* ecotype Col-0. Relative levels of *DOF1.7* transcripts in CMV(Ho)tr-inoculated and mock-inoculated Col-0 leaves (leaf/DOF1.7) **(A)** and *CBP1* transcripts in CMV(Ho)tr-inoculated and mock-inoculated Col-0 main roots (root/CBP1) **(B)** were measured by qPCR. The plants were treated with distilled water (DW) or NaCl (NaCl). The average relative amounts of *DOF1.7* or *CBP1* transcripts in CMV(Ho)tr-inoculated samples from three independent samples are shown by a gray bar chart with error bars (*n* = 3, SD), and their levels in mock-inoculated samples from three independent samples are indicated by a white bar chart with error bars (*n* = 3, SD). The different letters indicate a statistically significant difference in the average of relative amounts of *DOF1.7* or *CBP1* transcript (Turkey’s test, *p* < 0.05).

*DOF1.7* expression was downregulated in CMV(Ho)tr-inoculated leaves in comparison with mock-inoculated leaves (Figure 13A), but *CBP1* expression was upregulated in CMV(Ho)tr-infected roots as compared with the roots of mock-inoculated plants (Figure 13B). Furthermore, in CMV(Ho)tr-infected plants treated with 150 mM NaCl, the level of *DOF1.7* downregulation and *CBP1* upregulation was further enhanced (Figure 13).

## Discussion

### Infection With CMV(Ho) in *Arabidopsis halleri* and *Arabidopsis thaliana*

CMV(Ho) was isolated from *A. halleri*, which is a perennial wild weed and often naturally inhabits at a mineral land (Hohmann et al., 2014; Šrámková-Fuxová et al., 2017; Honjo and Kudoh, 2019). The analysis of CMV(Ho) RNA molecules indicated that, in addition to CMV genomic RNAs (RNA1, RNA2, and RNA3), CMV(Ho) contained DI-RNA3, in which the *3a* gene was partially or completely deleted, as well as a sat-RNA (Figure 2A and Supplementary Figure 2). DI-RNAs have been identified in the genomes of CMV and other RNA viruses (Graves and Roossinck, 1995; Simon et al., 2004; Pathak and Nagy, 2009). In most cases, the presence of DI-RNA modulates symptom severity in virus-infected host plants by reducing virus multiplication (Pathak and Nagy, 2009). sat-RNAs of CMV either enhance or suppress symptoms induced during co-infection with CMV (Hu et al., 2009). The nucleotide sequence of sat-RNAs clearly indicates its contribution to symptom modulation (Shimura et al., 2011; Smith et al., 2011). In *A. halleri* and *A. thaliana*, the level of CMV(Ho) multiplication significantly decreased in comparison with two virulent CMV strains (Figures 1C, 2B and Supplementary Figure 6). Moreover, while CMV(Ho)tr, only containing the tripartite CMV(Ho) genomic RNA1, RNA2, and RNA3, could multiply at a similar level as CMV(Y) (Figures 2B, 3C), co-infection of CMV(Ho)tr with DI-RNA3 or sat-RNA reduced the level of virus multiplication (Figure 2 and Supplementary Figure 8A). Lower virus multiplication may have a more negative effect for the virus, as it reduces the rate of virus transmission by aphid. Indeed, the amount of virus in the infected host plants is generally linked with the aphid transmission. However, standing on the view of the long-term symbiotic interaction between CMV(Ho) and *A. halleri*, lower virus multiplication seems to contribute to enlarge the infection period for the virus. It seems to be necessary to conduct further experiments to make clear the role of DI-RNA3 and sat-RNA on the interaction of CMV(Ho) and *A. halleri*.

Interestingly, infection with CMV(Ho)tr did not induce clear symptoms in *A. thaliana* and *A. halleri*, whereas CMV(Y) induced yellowing and stunting symptoms, although the levels of virus multiplication of CMV(Ho)tr and CMV(Y) were quite similar (Figures 2, 3 and Supplementary Figure 6). In many cases of a persistent viral infection of host plants, the virus multiplies at lower levels without clear symptoms. However, *A. halleri* and *A. thaliana* infected with CMV(Ho)tr did not develop any symptoms, and the asymptomatic infection was also observed in the CMV(Ho)tr-infected plants, although the level of CMV(Ho)tr was similar with that of a virulent strain. Whether the status of non-attenuated virus multiplication without developing symptoms has helped the virus, as a first step, to evolve and establish a persistent infection in the perennial *A. halleri* remains to be answered.

### The Possible Beneficial Influence of CMV(Ho)tr Infection in *Arabidopsis thaliana*

CMV(Ho)tr did not induce any symptoms in mature *A. thaliana*, but interestingly, it influenced plant growth and root development in very young *A. thaliana* (Figures 4, 5). The vegetative stage of plant growth of CMV(Ho)tr-infected plants was maintained for longer than control mock-inoculated *A. thaliana* (Figures 4A,B). This could be interpreted by the delaying of senescence in CMV(Ho)tr-infected plants in comparison with control plants (Figure 4C). This is consistent with the delayed flowering phenotype in *A. thaliana* infected with CMV (Pagán et al., 2007, 2008; Hily et al., 2014). They also reported age-dependent effects of CMV infection on the growth of *A. thaliana*. Moreover, the pattern of root development of CMV(Ho)tr-infected plants was changed from the dominancy of lateral root development to main root development (Figure 5). These facts suggest that persistent infection of the annual *A. thaliana* with CMV(Ho)tr might modulate plant growth and development in an age-dependent manner, because the physiological state of the host plants seems to be changed with their growth and development. However, it remains to be investigated whether the phenotypes are beneficial to the life cycle of plants in nature. The persistent viral infections of host plants occur much more common in the natural environment than is known. It is not unlikely that there is a favorable selection pressure for the presence and maintenance of persistent viral infections when these coincide with survival advantages of host plants *via* the modulation of plant growth and root development.

### Viral Determinant for Persistent Infection of *Arabidopsis thaliana* With CMV(Ho)

Although DI-RNA3 and sat-RNA did not seem to determine the status of symptomless infection with CMV(Ho), the analysis of a series of reassortant CMVs between CMV(Ho)tr and virulent CMV(Y) indicated that the determinant for the symptomless infection of CMV(Ho) is located on RNA2 of CMV(Ho) (Figures 6, 7). CMV(Ho) 2b protein, in which two amino acids were substituted from CMV(Y) 2b protein, still exhibited RNA-silencing suppressor activity, similar to CMV(Y) 2b protein (Figure 8 and Supplementary Figure 9). The two amino acid substitutions involved were neither located in the small interfering (si)RNA-binding domain, nucleo-cytoplasmic shuttling domain, nor at the phosphorylation site (KSPSE) in 2b protein (Lewsey et al., 2009, 2010a). The location of the two amino acid substitutions at positions other than those functional domains in 2b is in agreement with its preserved RNA-silencing suppressor activity. The two amino acid substitutions in CMV(Ho) 2b protein, which conferred symptomless infection (Figures 9, 10 and Supplementary Figure 11), were located at the N- and C-terminal variable regions of 2b protein, which seem to be associated with the induction or attenuation of symptom severity. To date, besides virus-induced RNA-silencing suppressor activity and symptom expression, the 2b protein is thought to associate with phenotypical changes of host plants, such as drought tolerance, interference with salicylic acid-mediated virus resistance, and disruption of jasmonic acid-mediated gene expression (Xu et al., 2008; Westwood et al., 2012). Accordingly, it influences CMV transmission by aphids, induces necrosis by inhibiting H_2_O_2_ scavenger catalase 3 activity (Ji and Ding, 2001; Lewsey et al., 2010b; Inaba et al., 2011; Westwood et al., 2012), and exerts multiple functions in host plants through its interaction with siRNA duplexes or AGO proteins (Zhang et al., 2006; Goto et al., 2007; Lewsey et al., 2007, 2010b; González et al., 2010; Duan et al., 2012; Hamera et al., 2012). The results from this study again provide support for 2b protein playing a role in disease symptomatology and, in the case of CMV(Ho), leading to a symptomless disease and thereby possibly, although speculative, supporting the establishment of a persistent viral infection in wild plants.

### The 2b Protein of CMV(Ho) Modulates Cytosine Hypomethylation Levels in the Promoter Regions of Genes in the *Arabidopsis thaliana* Genome

In *A. thaliana* ecotype Col-0 infected with CMV(Ho), the 2b protein interacted with AGO4 protein (Figure 11 and Supplementary Figure 13). An interaction between the 2b protein of virulent CMV strains and AGO proteins (AGO1 and AGO4) has been shown earlier in the host plants showing symptoms (Zhang et al., 2006; González et al., 2010; Duan et al., 2012; Hamera et al., 2012). For virulent CMV strains, their 2b proteins might contribute to the suppression of the RNA-silencing system through their interaction with AGO1 (Zhang et al., 2006) and induce symptom development, which seemed to be the result of blocking AGO1 cleavage activity and affecting microRNA metabolism in CMV-infected plants (Zhang et al., 2006). In our study, CMV(Ho) 2b protein interacted with AGO4, but not AGO1, in symptomless *A. thaliana* (Figure 11 and Supplementary Figures 12, 13). Thus, we hypothesize that CMV(Ho) 2b protein might modulate the function of AGO4, which is not related to the appearance of symptoms but is affecting the cytosine methylation level and leading to persistent infection of plants. It has been suggested that the direct interaction of 2b protein of virulent CMV strains with AGO4 protein of *A. thaliana* could counteract AGO4-related functions and reduce host genome methylation, resulting in symptom appearance (Hamera et al., 2012). Moreover, the analysis of single-base resolution methylomes of the genomic DNA of CMV-infected *Nicotiana tabacum* revealed that dynamic methylation of cytosine residues in CHH sequences occurs in the leaves of virulent CMV-infected plants at the symptom recovery stage (Wang et al., 2018). Indeed, the involvement of AGO4 in the regulation of the cytosine hyper/hypomethylation levels of host genomic DNA, thereby modulating host gene expression, has been well demonstrated (Bies-Etheve et al., 2009; He et al., 2009; Ye et al., 2016). Interestingly, in whole-genome bisulfite sequencing analysis, the promoter regions of 82 genes were hypomethylated in CMV(Ho)tr-infected plants in comparison with control plants (Supplementary Figure 15 and Supplementary Table 7), but without inducing symptoms. It remains to be investigated whether the changes in gene expression under the control of the promoter regions of these 82 genes also modulate downstream gene expression networks in plants infected with CMV(Ho), which could possibly contribute to a persistent infection and/or modulate other parts of its life cycle of plants.

In our Co-IP experiment, the interaction of 2b protein of CMV(Ho) with AGO5 was also observed. The role of the interaction of 2b protein with AGO5 will be a subject of future research.

### The Impact of a Persistent Viral Infection on the Life Cycle of Wild Plants

To further confirm whether CMV(Ho) 2b protein confers hypomethylation of the promoter regions of 82 genes, *DOF1.7* and *CBP1* were chosen as representative genes because they both encode transcriptional regulators, and changes in their expression seem to modulate diverse downstream signaling pathways. The plant-specific DOF transcription factor was identified initially in maize and contained a conserved zinc finger domain that binds specifically to DNA with the 5′-(A/T)AAG-3′ core sequence (Yanagisawa and Sheen, 1998; Hir and Bellini, 2013). Regarding *DOF1.7*, it has been shown to be potentially expressed in vascular tissues at a specific step of root and leaf development by transcriptome analysis of microdissected provascular/procambial cells or complete vascular bundles (Gandotra et al., 2013), and its overexpression in transgenic tobacco enhances nitrogen assimilation under low-nitrogen conditions (Wang et al., 2013). CBP1 (also known as MEE14) has been shown to interact with CCG, the mediator subunits MED7 and MED9, and the C-terminal domain of the NPRB1 subunit of RNA polymerase II and to recruit AGAMOUS-like transcription factors to promote the expression of target genes (Li et al., 2015). *CBP1* is expressed in vegetative tissues and central cells. *CBP1* expression is reported to be upregulated in epidermal cells of the radial zone of roots in response to salt stress (Dinneny et al., 2008).

The hypomethylation of the promoter regions of *DOF1.7* and *CBP1* in CMV(Ho) *2b*-transformed plants was confirmed directly by bisulfite sequencing (Figure 12 and Supplementary Figures 16, 17). Intriguingly, salt stress-responsive downregulation of *DOF1.7* expression and upregulation of *CBP1* expression are further enhanced in CMV(Ho)tr-infected plants exhibiting no symptoms (Figure 13). Hence, 2b-mediated cytosine hypomethylation in the promoter regions of the genomic DNA of host plants may enable host plants to modulate gene expression in response to salt stress. Although the change of *DOF1.7* and *CBP1* expression by CMV(Ho)tr infection is an example of the co-relationship between hypomethylation and the change of gene expression in plants persistently infected with CMV(Ho), it remains to be elucidated whether the same event may be applied to other genes in which their promoter regions were hypomethylated.

The enhanced change of *CBP1* and *DOF1.7* expression patterns in CMV(Ho)tr-infected plants was correlated with CMV(Ho) 2b protein-mediated cytosine hypomethylation in their promoter regions. This observation suggests that the status of host gene expression may be epigenetically primed to respond immediately or strongly to some types of abiotic environmental stresses by 2b protein-mediated cytosine hypomethylation in the promoter regions of the host genome. It is, therefore, not unlikely that persistent viral infections may have a much more complicated and major impact on the life cycle of wild and perennial plants in the natural environment.

## Data Availability Statement

The GenBank/EMBL/DDBJ accession numbers for cDNAs to CMV(Ho)RNA1, CMV(Ho)RNA2, CMC(Ho)RNA3, CMV(Ho)RNA3.DI-1, CMV(Ho)RNA3.DI-6, and CMV(Ho) sat-RNA are LC593244, LC593245, LC593246, LC593247, LC593248, and LC593249 respectively. The data for whole genome bisulfate sequencing have been deposited in the DDBJ Sequence Read Archive (DRA) (https://www.ddbj.nig.ac.jp/dra/index-e.html) and are accessible through DRR Run accession number: DRR311730-DRR311732 (ht600414_ht-0001_Run_0001-0005).

## Author Contributions

HT, TF, and RK conceived the study. HT conducted the experiments and wrote the original draft. HT, MT, SM, and SK analyzed the data. HT, SM, SA, YK, TF, and RK interpreted the results. RK and HT reviewed and edited the manuscript. All authors contributed to the article and approved the submitted version.

## Conflict of Interest

The authors declare that the research was conducted in the absence of any commercial or financial relationships that could be construed as a potential conflict of interest.

## Publisher’s Note

All claims expressed in this article are solely those of the authors and do not necessarily represent those of their affiliated organizations, or those of the publisher, the editors and the reviewers. Any product that may be evaluated in this article, or claim that may be made by its manufacturer, is not guaranteed or endorsed by the publisher.
