# Supplementary material for: Cucumber Mosaic Virus Infection in Arabidopsis: A Conditional Mutualistic Symbiont?
Source: Front Microbiol. 2022 Jan 7;12:770925. doi: 10.3389/fmicb.2021.770925 (PMC8776717; doi:10.3389/fmicb.2021.770925)
Supplement: Supplementary file 1 [file Data_Sheet_1.docx]

**SupplementaryTableS2|** List of primers for amplification of the DIG-labelling probes by PCR to detect CMV(Ho) RNAs in this study.

**SupplementaryTableS3|** List of primers to construct the *in vitro* transcription vectors for infectious CMV RNAs in this study.

**SupplementaryTableS4|** List of primers for the construction of the binary vectors expressing 2b for CMV(Ho) RNA2 and AGOs under the control of the CaMV35S promoter in this study.

**SupplementaryTableS5|** Primers for bisulfite sequencing performed in this study.

**SupplementaryTableS6|** Primers for gene expression analysis by qPCR performed in this study.

**SupplementaryTable S7 |** Genes in which the promoter region was hypomethylated in CMV(Ho)tr-inoculated *A. thaliana* ecotype Col-0 leaves.

**SupplementaryFigure S1 |** Schematic structures of *in vitro* transcription vectors for infections with CMV RNAs. RNA1, RNA2, and RNA3 of CMV(Ho) and CMV(Y); DI-RNA3 of CMV(Ho); CMV(Ho) sat-RNA; chimeric CMV RNA2 in which two ORFs were reciprocally exchanged between CMV(Ho) and CMV(Y), were cloned under the control of the T7 promoter (T7-pro) in pUC18. Each vector name is shown on the left.A *Not*I restriction site was located at the 3′-region of each cDNA to linearize the vector plasmid as a template for *in vitro* transcription. Blue rectangle indicates the protein-coding region of CMV(Ho), and red rectangle indicates that of CMV(Y). Since CMV sat-RNA does not encode any protein, its cDNA is shown as a thick black line.

**Supplementary Figure S2 |**Schematic structures of cDNAs for DI-RNA3. CMV(Ho) RNA3.DI-1 and CMV(Ho) RNA3.DI-6 are shown based on the nucleotide sequences of the smaller RNA3 cDNA products. Protein-coding region is shown as a blue rectangle, and the defective region in RNA3 is indicated by a white rectangle with nucleotide sequence positions of full-length RNA3. The 5′-end of each RNA has a ^7^mGpppG-cap structure (Cap), and the 3′-end of each RNA has the same nucleotide sequence (5′-CCA-3′).

**SupplementaryFigure S3** | Schematic structures of *in vitro* transcription vectors for infection with CMV RNA2 carrying one or two nucleotide substitutions resulting in amino acid substitutions in 2b protein. cDNAs for CMV RNA2 carrying one or two nucleotide substitutions resulting in amino acid substitutions in 2b protein were cloned under the control of the T7 promoter (T7-pro) in pUC18. Each vector name is shown on the left.A *Not*I restriction site was located at the 3′-region of each cDNA to linearize the vector DNA plasmid as a template for *in vitro* transcription. Blue lines indicate deduced amino acid substitutions: A21V, S77L, and A106V.

**SupplementaryFigure S4** | Schematic structures of binary vectors for the expression of *2b* cDNA from CMV(Ho) or CMV(Y) and *A. thaliana AGO1-10* cDNAs in plant cells. (**A**) CMV(Ho) *2b* cDNA, CMV(Y) *2b* cDNA, or CMV(Y) *2b* cDNA containing nucleotide substitutions changing two amino acids (S77L/A106V or A21V/S77L) in 2b protein were cloned between the CaMV 35S promoter (35S-pro) and NOS terminator (NOS-term) of pRI201AN. GFP was also cloned between 35S-pro and NOS-term of pRI201AN. Each vector name is shown on the left side. Triangles indicate the right border (RB) and left border (LB) sequences derived from Agrobacterium T-DNA. (**B**) *2b* cDNA of CMV(Ho) or CMV(Y) with the FLAG epitope sequence at the 3′-terminus and *A. thaliana AGO1-10* cDNAs with the HA epitope sequence at the 5′-terminus were cloned into the same site of pRI201AN, respectively.

**SupplementaryFigure S5|** Survey of CMV infection in naturally growing *A. halleri* at an abandoned “Hosokura” mine in the Tohoku region of Japan in April, 2017. Photographs of the plant community of *A. halleri* (**A**), an *A. halleri* plant (**B**), and enlarged photograph of *A. halleri*(**C**). CMV coat protein in symptomless plants derived from the independent plant community was detected by western blotting and a part of the data is shown in the panel (**D**). RuBisCO band, which was detected by CBB staining, is shown as an internal control.

**SupplementaryFigure S6|** *Arabidopsishalleri*infected with CMV(Ho), CMV(Ho)tr or virulent CMV(Y). At 21 dpi with CMV(Ho), *A. halleri* plants [CMV(Ho), CMV(Ho)tr and CMV(Y)], which were infected with CMV(Ho), CMV(Ho)tr, and virulent CMV(Y), respectively, were photographed (**A**). As a control, *A. halleri* plants (Mock)were rub-inoculated with 10 mM sodium phosphate buffer (pH 7.5). Virus infection was analyzed by detecting CMV coat protein in non-inoculated upper leaves at 21 dpi by western blotting (**B**). Four plants were inoculated with each virus and the representative data was shown. The RuBisCO band, which was detected by CBB staining, is shown as an internal control.

**Supplementary Figure S7**| Phylogenetic tree analysis of deduced amino acid sequences of the coat protein of CMV(Ho) with those of 11 other CMV strains. To classify CMV(Ho), the phylogenetic relationship based on the deduced amino acid sequence of the coat protein was determined between CMV(Ho) and 11 other CMV strains: CMV(Y), CMV(As), CMV(ND1), CMV(ND2) and CMV(Fny) in subgroup IA; CMV(M), CMV(KS44), CMV(P6), and CMV(FC) in subgroup IB; and CMV(Q) and CMV(TN). The amino acid sequence of the coat protein of peanut stunt virus (PSV) strain ER was used as an outgroup. The tree was created based on the Neighbor-Joining method using MEGA11. The percentage of replicate trees in which the associated taxa clustered together in the bootstrap test (1,000 replicates) are shown next to the branches The tree is drawn to scale, with branch lengths (next to the branches) in the same units as those of the evolutionary distances used to infer the phylogenetic tree. The bar below the tree indicates the number of nucleotide substitutions per site. The caption of “CMV(Ho)” was enclosed by red line.

**SupplementaryFigure S8|** Influence of CMV(Ho) DI-RNA3 and sat-RNA on CMV(Ho)tr multiplication. Fully expanded leaves of *A. thaliana* ecotype Col-0 were inoculated with CMV(Ho)tr generated by infection with a mixture of *in vitro* transcribed CMV(Ho) RNA1, RNA2, and RNA3, CMV(Ho)tr plus *in vitro* transcribed CMV(Ho) sat-RNA [CMV(Ho)tr+sat-RNA], or CMV(Ho)tr plus *in vitro* transcribed RNA3.DI-6 [CMV(Ho)tr+RNA3.DI-6], and virus multiplication was analyzed by immunologically detecting coat protein (indicated by red arrow) at 14 dpi (**A**). As an internal control, ribulose-1,5-bisphosphate carboxylase/oxygenase (RuBisCO) was detected by Coomassie Brilliant Blue G250 (CBB) staining. The amount of full length CMV(Ho) RNA3 was measured by quantitative PCR (qPCR) (**B**). The qPCR was done using a set of specific primers which do not bind to either RNA3.DI-6 or sat-RNA. Average of relative amounts of full length CMV(Ho) RNA3 in CMV(Ho)tr, CMV(Ho)tr+RNA3.DI-6 or CMV(Ho)tr+sat-RNA-inoculated leaves from three independent plants (n=3) isshown by a gray bar chart with error bars (SD). The different letters indicate a statistically significant difference in the average of relative amounts of full length CMV(Ho) RNA3 (Turkey’stest, *p*< 0.05).Infection of virus-inoculated leaves with CMV(Ho), CMV(Ho) sat-RNA, or RNA3.DI-6 was detected by RT-PCR (**C**). Each cDNA fragment was synthesized by RT-PCR using a set of primers specific to RNA3 or sat-RNA with total RNA isolated from leaves inoculated with viruses as a template: CMV(Ho)tr generated by infection with a mixture of *in vitro* transcribed CMV(Ho) RNA1, RNA2, and RNA3, CMV(Ho)tr plus *in vitro* transcribed CMV(Ho) sat-RNA, or CMV(Ho)tr plus *in vitro* transcribed RNA3.DI-6,and analyzed by agarose gel electrophoresis.

**SupplementaryFigure S9|** Quantitative analysis of the RNA-silencing suppressor activity of 2b proteins of CMV(Ho) and virulent CMV(Y) and 2b proteins of CMV(Y) carrying two amino acid substitutions using *N. benthamiana* 16c. The binary vectors pRI201AN::2b.Y(S77L/A106V), pRI201AN::2b.Y(A21V/S77L), pRI201AN::2b.Y, pRI201AN::2b.Ho, or pRI201AN (**SupplementaryFigure S4**) were transiently co-expressed with 35S::GFP in the leaves of *N. benthamiana* 16c, in which RNA silencing of GFP expression in induced, by the agroinfiltration method. As a control, pRI201AN was only agroinfiltrated. The amounts of GFP in the leaves of *N. benthamiana* 16c transiently co-expressing 35S::GFP with pRI201AN::2b.Y, pRI201AN::2b.Ho, or pRI201AN, were quantitatively measured by ELISA using antibody against GFP (**A**). The relative amounts of GFP in the leaves of *N. benthamiana* 16c transiently co-expressing 35S::GFP with pRI201AN::2b.Y(S77L/A106V), pRI201AN::2b.Y(A21V/S77L), or pRI201AN, were also quantitatively done by ELISA (**B**). Average of relativeamounts of GFP of the infiltrated leaves (n = 3) is shown by bar chart with error bars (SD). The different letters indicate a statistically significant difference in the average of relative amounts of GFP (Turkey’stest, *p*< 0.05).

**SupplementaryFigure S10|** Detection of the accumulation of 2b proteins of CMV(Ho) and virulent CMV(Y), and 2b proteins of CMV(Y) carrying two amino acid substitutions in agroinfiltrated leaves of *N. benthamiana* 16c. The binary vectors pRI201AN::2b.Y(S77L/A106V), pRI201AN::2b.Y(A21V/S77L), pRI201AN::2b.Y, pRI201AN::2b.Ho, or pRI201AN (**SupplementaryFigure S4A**) were transiently co-expressed with 35S::GFP in the leaves of *N. benthamiana* 16c, in which RNA silencing of GFP expression in induced, by the agroinfiltration method. As a control, pRI201AN was only agroinfiltrated. The accumulation of 2b protein in the leaves transiently co-expressing 35S::GFP with pRI201AN::2b.Y, pRI201AN::2b.Ho, or pRI201AN, was detected immunologically using an antibody against the HA epitope (**A**). The accumulation of 2b protein in the leaves transiently co-expressing 35S::GFP with pRI201AN::2b.Y(S77L/A106V), pRI201AN::2b.Y(A21V/S77L), pRI201AN::2b.Y, or pRI201AN, was detected immunologically using an antibody against the HA epitope (**B**). As an internal control, RuBisCO was detected by Ponceau-S staining and shown in the lower panel.

**SupplementaryFigure S11 |** *Arabidopsis thaliana* ecotype Col-0 expressing FLAG epitope-tagged 2b protein of CMV(Ho) or virulent CMV(Y). Col-0 plants were transformed with p35S::2b.Ho-FLAG or p35S::2b.Y-FLAG. Representative each transgenic plants carrying homozygous transgene, was photographed at 3 months after sowing (**A**). As a control, Col-0 plants were transformed with the binary pRI201AN vector. Each transformant was named Col::2b.Ho-FLAG, Col::2b.Y-FLAG, or Col::vector (Control). Accumulation of 2b protein of CMV(Ho) or CMV(Y) was detected immunologically by western blotting using an antibody against the FLAG epitope (α-FLAG) (**B**). In five independent transgenic lines of Col::2b.Ho-FLAG (#10-14) and those of Col::2b.Y-FLAG (#1-5), FLAG epitope-tagged 2b protein was analyzed. As an internal control, RuBisCO was detected by Ponceau-S staining.

**SupplementaryFigure S12|** Analysis of the interaction of CMV(Ho) 2b protein with AGO1-5 by co-immunoprecipitation (Co-IP). For Co-IP, HA epitope-tagged *AGO* cDNA (p35S::HA-AGO) (**SupplementaryFigure S4B**) and FLAG epitope-tagged *2b* cDNA of CMV(Ho) (p35S::2b.Ho-FLAG) (**SupplementaryFigure S4B**) were transiently co-expressed in *N. benthamiana* leaves by the agroinfiltration method.HA-tagged AGO1-5 (HA-AGO1, HA-AGO2, HA-AGO3, HA-AGO4, or HA-AGO5) and FLAG-tagged 2b protein (2b.Ho-FLAG) in leaf homogenates from *N. benthamiana* leaves were used as “Input” for Co-IP analysis. AGO1-5 proteins (HA-AGO1, HA-AGO2, HA-AGO3, HA-AGO4, or HA-AGO5) were detected immunologically by western blotting using an antibody against the HA epitope (α-HA) (**A** and **C**), and CMV(Ho) 2b protein (2b.Ho-FLAG) was detected using an antibody against the FLAG epitope (α-FLAG) (**B** and **D**).

**SupplementaryFigure S13 |** Co-immunoprecipitation (Co-IP) analysis of AGO4 protein with 2b protein of CMV(Ho). Three lines of *A. thaliana* ecotype Col-0 (lines #1, #2, and #3) were transformed with HA epitope sequence-tagged cDNA for *AGO4* mRNA and FLAG epitope sequence-tagged cDNA for *2b* RNA of CMV(Ho) (p35S::2b.Ho-FLAG and p35S::HA-AGO4 in**SupplementaryFigure S4B**) and named Col::35Spro.2b.Ho-FLAG/35Spro.HA-AGO4 (lines #1, #2, and #3). As a control, three lines of Col::35Spro.HA-AGO4 plants (lines #1, #2, and #3), which were transformed with HA epitope sequence-tagged cDNA for *AGO4* mRNA, and three lines of Col::35Spro.2b.Ho-FLAG plants (lines #11, #12, and #13 in **Figure 10**), which were transformed with FLAG epitope sequence-tagged cDNA for *2b* RNA of CMV(Ho), were used. HA-tagged AGO4 protein (HA-AGO4) and FLAG-tagged2b protein (2b.Ho-FLAG) in leaf homogenates from the transgenic Col-0 plants, which were used as “Input” for Co-IP analysis, were detected immunologically using antibodies against the HA (α-HA) (**A**) and FLAG (α-FLAG) epitopes (**B**), respectively. In Co-IP, HA-AGO4 binding to anti-HA agarose beads was detected immunologically by α-HA (**C**), and 2b-FLAG, which was co-immunoprecipitated with HA-AGO4, was assessed by α-FLAG (**D**).

**Supplementary Figure S14 |**Distribution of cytosine methylation levels throughout the genome between CMV(Ho)tr- vs. mock-inoculated leaves of *A. thaliana* ecotype Col-0. The plots show the distribution of the methylation level of CG, CHG, and CHH in whole genomic DNA between CMV(Ho)tr- (red bar) and mock (blue bar)-inoculated leaves.

**SupplementaryFigure S15**| Mapping of promoter regions in which cytosine hypomethylation levels increased in CMV(Ho)tr-inoculated leaves in comparison with mock-inoculated leaves on five chromosomes. The ratio (≥0.2) of hypomethylated cytosine in CG, CHG, and CHH in promoter regions in CMV(Ho)tr-inoculated leaves, but not in mock-inoculated leaves, to all cytosine sites in the corresponding genomic regions, was selected for genes carrying cytosine hypomethylation in their promoter region after CMV(Ho)tr infection, and their AGI codes were mapped on five chromosomes. Two promoter regions, which were located upstream of genomic regions encoding the transcriptional regulators *DOF1.7* (AT1G5170) and *CBP1* (AT2G15890) are shown by red characters and were used for further investigations of their response to CMV(Ho)tr infection.

**SupplementaryFigure S16 |** Schematic diagram of the promoter region of *DOF1.7* showing the positions of hypomethylated cytosines in *A. thaliana* ecotype Col-0 transformed with FLAG epitope sequence-tagged cDNA for the *2b* gene of CMV(Ho) or vector control. The promoter region was shown blue arrow and DOF1.7-coding region was done by blue square. Degree of methylation in cytosines of the hypomethylated promoter region of *DOF1.7* in CMV(Ho) *2b*-transformed Col-0 (Col::35Spro.2b.Ho-FLAG) and control Col-0 (vector control) plants was visualized by bisulfite sequencing analysis of the promoter regions of 10 independent clones. The positions of cytosine hypermethylation sites in CHH are shown by solid green circles, and cytosine hypomethylated sites in CHH are indicated by empty green circles. The positions of cytosine hypermethylation sites in CG are shown by solid red circles, and cytosine hypomethylated sites in CG are indicated by empty red circles.

**SupplementaryFigure S17 |** Schematic diagram of the promoter region of *CBP1* showing the positions of hypomethylated cytosines in *A. thaliana* ecotype Col-0 transformed with FLAG epitope sequence-tagged cDNA for the *2b* gene of CMV(Ho) or vector control. The promoter region was shown blue arrow and CBP1-coding region was done by blue square with gray color intron. Degree of methylation in the cytosine hypomethylated promoter region of *CBP1* in CMV(Ho) *2b*-transformed (Col::35Spro.2b.Ho-FLAG) and control Col-0 (vector control) plants was visualized by bisulfite sequencing analysis of the promoter regions of 10 independent clones. Positions of cytosine hypermethylation sites in CHH are shown by solid green circles, and cytosine hypomethylated sites in CHH are indicated by empty green circles. The positions of cytosine hypermethylation sites in CG and CHG are shown by solid red and blue circles, respectively, and cytosine hypomethylated sites in CG and CHG are indicated by empty red and blue circles, respectively.
